# An 18 gene expression-based score classifier predicts the clinical outcome in stage 4 neuroblastoma

**DOI:** 10.1186/s12967-016-0896-7

**Published:** 2016-05-17

**Authors:** Daniela Formicola, Giuseppe Petrosino, Vito Alessandro Lasorsa, Piero Pignataro, Flora Cimmino, Simona Vetrella, Luca Longo, Gian Paolo Tonini, André Oberthuer, Achille Iolascon, Matthias Fischer, Mario Capasso

**Affiliations:** Dipartimento di Medicina Molecolare e Biotecnologie Mediche, Università degli Studi di Napoli Federico II, 80145 Naples, Italy; CEINGE Biotecnolgie Avanzate Scarl, Naples, Italy; Department of Oncology, Santobono-Pausilipon Children’s Hospital, Naples, Italy; U.O.C. Bioterapie, IRCCS AOU San Martino-IST, National Cancer Research Institute, Genoa, Italy; Laboratory of Neuroblastoma, Onco/Hematology Department SDB University of Padua, Pediatric Research Institute, Padua, Italy; Department of Pediatric Oncology and Hematology, and Center for Molecular Medicine Cologne (CMMC), University of Cologne Children’s Hospital, Cologne, Germany; Max Planck Institute for Metabolism Research, Cologne, Germany

**Keywords:** Neuroblastoma, Risk score, Prognosis, Microarray

## Abstract

**Background:**

The prognosis of children with metastatic stage 4 neuroblastoma (NB) has remained poor in the past decade.

**Patients and methods:**

Using microarray analyses of 342 primary tumors, we here developed and validated an easy to use gene expression-based risk score including 18 genes, which can robustly predict the outcome of stage 4 patients.

**Results:**

This classifier was a significant predictor of overall survival in two independent validation cohorts [cohort 1 (n = 214): *P* = 6.3 × 10^−5^; cohort 2 (n = 27): *P* = 3.1 × 10^−2^]. The prognostic value of the risk score was validated by multivariate analysis including the established markers age and *MYCN* status *(P* = 0.027). In the pooled validation cohorts (n = 241), integration of the risk score with the age and/or *MYCN* status identified subgroups with significantly differing overall survival (ranging from 35 to 100 %).

**Conclusion:**

Together, the 18-gene risk score classifier can identify patients with stage 4 NB with favorable outcome and may therefore improve risk assessment and treatment stratification of NB patients with disseminated disease.

**Electronic supplementary material:**

The online version of this article (doi:10.1186/s12967-016-0896-7) contains supplementary material, which is available to authorized users.

## Background

Neuroblastoma (NB) is the most frequent solid tumor of early childhood with a remarkable variation in clinical presentation ranging from favorable localized tumors that can spontaneously regress to metastatic disease with unfavorable outcome [1]. Within the cohort of patients with disseminated disease the International Neuroblastoma Staging System (INSS) separates unfavorable stage 4 NB, which comprises about 45–50 % of the cases and is defined as a primary tumor with dissemination to distant lymph nodes, bone, bone marrow, liver, skin, or other organs [2], from favorable stage 4s (special) disease.

A recent review study on 11,037 children with NB from Australia, Europe, Japan, North America has shown that, during the period between 1974 and 2002 the event-free survival of stage 1, 2, 3 and 4 s patients has consistently increased while it hardly changed for stage 4 patients [3]. Another paper reviewed the clinical and survival data of 2216 children with NB enrolled in the Italian Neuroblastoma Registry over a 27-year period (1979–2005). From 1992 to 2005, the overall survival (OS) of patients with stage 3 significantly improved from 67.3 to 88.5 %, whereas the OS of stage 4 patients increased only by 3 % (26–29 %) [4]. These findings demonstrate that no substantial progress in survival has been made for stage 4 patients.

To date, age and *MYCN* status remain the most important markers of outcome in patients with stage 4 NB. Patients ≥18 months of age with stage 4 NB and those with *MYCN*-amplified stage 4 disease are defined as “high-risk” [1]. They are usually sensitive to dose-intensive chemotherapy: a majority of patients achieve remission after induction chemotherapy, surgery and radiotherapy, but most patients relapse even with consolidation therapy. Despite intensive multimodal treatment, these high-risk NB patients therefore have an OS of less than 40 % [1] and discrimination of ultra-poor outcome patients from those with a more favorable prognosis remains poor with current classification systems.

Gene expression profiling by means of microarrays [5, 6] has been shown to be useful in classifying tumors and predicting patient outcome in various types of cancer [7–9]. As such, numerous prognostic gene signatures have been developed to classify NB patients [10, 11]. In spite of the robustness of the published signatures in predicting NB outcome, so far none has been introduced into clinical risk stratification systems. This is probably due to various reasons such as: (i) most of the gene classifiers are built on heterogeneous patient cohorts without differentiating among INSS stage and other clinical and genetic markers, (ii) gene expression profiles can vary according to the microarray platform, (iii) analytic strategy used. Only two prognostic studies have been focused on the well-defined molecular and clinical subgroup of patients with metastatic NB lacking *MYCN* amplification [12, 13].

To overcome these limitations, we developed a robust and reproducible 18-gene expression based risk-scoring system able to predict OS of children with stage 4 NB by using a different and innovative strategy based on microarrays independent validations.

## Patients and methods

**Fig. 1:**
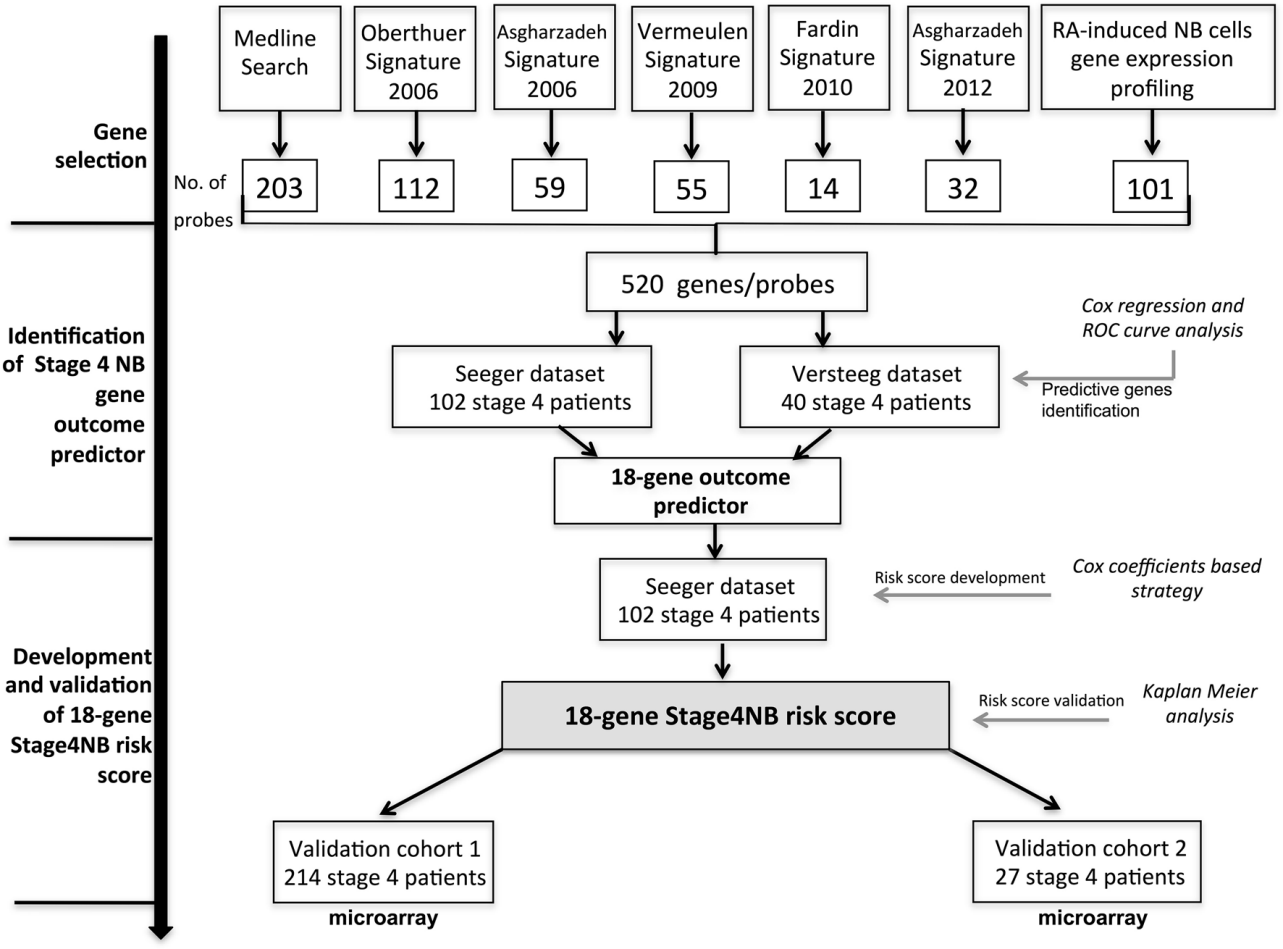
Outline of the strategy used for building and validating the gene-based scoring system of stage 4 neuroblastoma (NB). *Gene selection* The selected genes were obtained by (1) Medline Search using as keywords “Neuronal marker” and “Neuroendocrine marker” (2) published gene-signatures of NB; (3) analysis of publicly available microarray gene expression (GSE9169) on three NB cell lines treated with retinoic acid. *Identification of Stage 4 NB gene outcome predictor* freely downloadable gene expression datasets named Seeger dataset (GSE16254) and Versteeg dataset (GSE16476) have been used. *Development and validation of 18-gene Stage4NB risk score* we used the already published microarray gene expression data of 214 stage 4 cases (GSE45547) and new 27 stage 4 cases that have been deposited in GEO (Gene Expression Omnibus) database (GSE79910)

The analytic strategy is shown in Fig. 1. Our study has selected 520 unique probes for 426 genes as putative clinical markers based on the following sources:

- 203 markers by Medline search through the PubMed database (1990–2014) by using the keywords “Neuronal marker” and “Neuroendocrine marker”. 115 articles were selected on the basis of the best available evidence for the specific question;
- published gene-signatures
- 112 genes Oberthuer et al. [14]
- 55 genes Asgharzadeh et al. [12]
- 59 genes Vermeulen et al. [15]
- 32 genes Fardin et al. [16]
- 14 genes Asgharzadeh et al. [13];
- 101 genes/probes obtained by analysis of publicly available microarray of gene expression data (GSE9169) on different NB cell lines treated with retinoic acid (RA). This latter data were used to select genes involved in morphologic differentiation to ganglioneuromatous histopathology which are recognized as a positive prognostic sign in NB.

The pre-selection of 520 unique probes (Additional file 1: Tables S1, S2 and S3) is described in Additional file 1.

### Identification of the optimal gene set to predict overall survival

The optimal outcome predictor of stage 4 NB was built by using normalized gene expression array data of two independent sets of NB patients (n = 142):

1. “Seeger dataset” including 102 stage 4 samples downloaded from the website Oncogenomics (http://home.ccr.cancer.gov/oncology/oncogenomics/). Affymetrix HG-U133A and HG-U133B array (GSE16254);
2. “Versteeg dataset” including 40 stage 4 samples downloaded from the website R2: microarray analysis and visualization platform (http://hgserver1.amc.nl/cgi-bin/r2/main.cgi). Affymetrix HG-U133 Plus 2.0 array (GSE16476).

Development of the gene expression–based prognostic model is described in Additional file 1.

### Development of the 18-gene stage 4 NB (Stage4NB) risk score

We adopted a previously developed strategy using the Cox regression coefficient of each gene among 18-gene set from the Seeger cohort [17, 18]. The risk score for each patient was derived by multiplying the expression level of a gene by its corresponding coefficient (risk score = sum of Cox coefficient of Gene Gi X expression value of Gene Gi). Patients of Seeger dataset were dichotomized into both a high-risk group and a low-risk group, using the 50th percentile (median) cutoff of the risk score as the threshold value.

### Validation of the 18-gene stage 4 NB (Stage4NB) risk score score in independent test sets

Both the coefficient and the threshold value derived from the Seeger cohort were directly applied to the gene expression data from the exploration data set (Seeger cohort) and independent test sets of microarray experiments termed the “validation cohort 1” and “validation cohort 2” comprising gene expression microarray data of 214 (GSE45547) and 27 (GSE79910) stage 4 NB patients collected at University of Cologne Children’s Hospital in Germany (n = 20) and Gaslini Children Hospital in Italy (n = 7). Table 1 summarizes the features of patient cohorts. The latter set of 27 tumors has been newly analyzed for this study.

**Table 1:** Clinical features of stage 4 NB patients

Variables	Seeger cohort^a^	Versteeg cohort^b^	Validation cohort 1^c^	Validation cohort 2^d^
	n = 102	n = 40	n = 214	n = 27
Sex				
Male	N/A	24 (60 %)	122 (60.1)	13 (48.1)
Female	N/A	16 (40 %)	81 (39.9)	14 (51.8)
N/A			12	
Age				
Median (years)		2.5	2.7	2.6
<18 months	N/A	8 (20.0 %)	68 (31.8 %)	8 (29.6)
≥18 months	N/A	32 (30.0 %)	146 (68.2 %)	19 (70.4)
MYCN amplification				
Yes	0 (0 %)	15 (37.5 %)	68 (31.9 %)	14 (51.8)
No	102 (100 %)	25 (62.5 %)	145 (68.1 %)	13 (48.1)

*N/A* not available, *NB* neuroblastoma

^a,b,c^Microarray gene expression data downloaded from GEO datasets (GSE16254, GSE16476, GSE45547)

^d^Microarray gene expression data produced from a new cohort of stage 4 tumors (GSE79910)

### Microarray technology

RNA preparation was performed essentially as described previously [19]. Collection of patient data and samples was by the clinicians responsible for patient care, with written informed consent obtained from all children’s parents or legal guardians, and with approval by local university ethical committee (Ethical Committee of the University Federico II, C. Romano, Napoli, Italy). Subsequently, single-color gene-expression profiles were generated using customized 4 × 44 K oligonucleotide microarrays produced by Agilent Technologies (Palo Alto, CA, USA) as described previously [20]. More details are in Additional file 1.

### Statistical analysis

Kaplan–Meier estimates for OS were calculated and compared by log-rank test. Only death from disease was considered as an event. Cox regression models were applied using a stepwise selection procedure recommended by Collett [21] to analyze the prognostic value of potentially prognostic factors. Support Vector Machines (SVM)-based area under receiver operating characteristic (ROC) curve method as implemented in Gene Expression Model Selector (GEMS) software utilizing ten-fold cross-validation and linear polynomial kernel for SVM [22] was used to evaluate the performance of the published gene signatures and the 18-gene optimal outcome predictor. The gene network and gene ontology (GO) analysis was performed by the website GeneMANIA [23].

## Results

### Selection of 18 genes for predicting overall survival in stage 4 NB

The analytic approach based on a re-analysis of public data and the application of Cox regression and ROC curve method (Fig. 1; Additional file 1: Fig. S1 and S2) allowed us to identify a signature as predictor of overall survival (OS) composed of 20 probes for 18 unique genes for patients with stage 4 NB (Tables 1, 2). The predictor score that maximized the area under the curve (AUC) in the Seeger dataset (Additional file 1: Fig. S3) contained 15 genes (AUC = 0.94) whereas that in Versteeg dataset (Additional file 1: Fig. S3) contained 4 genes (AUC = 0.96). One gene (*FOXP1*) was shared between the two analyses. The distribution of the 18 genes according to the original source is reported in Additional file 1: Table S4. To evaluate the prognostic ability of each signature included in this study, we used SVM-based AUC analysis. The 18-gene signature predicted the OS of better than other published gene signatures [12–16] in both the Seeger (AUC = 94.63) and Versteeg (AUC = 88.33) datasets (Additional file 1: Table S5).

**Table 2:** Gene description and regression coefficients from Cox regression analysis

Symbol	Gene name	Chromosome^a^	Function	Coefficient
*ADCY1*	Adenylate cyclase 1 (brain)	7p12.3	Drug-target, membrane, signal transduction	−0.963
*AKR1C1*	Aldo–keto reductase family 1, member C1	10p15.1	Drug-target	−0.740^b^ and −0.681
*ARHGEF10L*	Rho guanine nucleotide exchange factor (GEF) 10-like	1p36.13	Signal transduction	−2.902
*BTBD3*	BTB (POZ) domain containing 3	20p12.2	–	0.807
*C9orf130*	Chromosome 9 open reading frame 130	9q22.32	–	0.612
*FOXP1*	Forkhead box P1	3p14.1	TF, transcription regulator activity	−0.984
*GFRA3*	GDNF family receptor alpha 3	5q31.2	Development, differentiation, membrane, signal transduction	−0.916
*GNAI1*	Guanine nucleotide binding protein	7q21.11	Membrane, signal transduction	−1.049^b^ and −1.48
*HOXC6*	Homeobox C6	12q13.3	TF, development, transcription regulator activity, transcriptional repressor activity	−0.786
*ING3*	Inhibitor of growth family, member 3	7q31.31	–	−0.918
*LOC153682*	Uncharacterized LOC153682	5p13.1	–	−0.886
*PGM2L1*	Phosphoglucomutase 2-like 1	11q13.4	–	−1.075
*RUNDC3B*	RUN domain containing 3B	7q21.12	–	−1.0156
*PRKACB*	Protein kinase, cAMP-dependent, catalytic, beta	1p31.1	Kinase, signal transduction	−1.071
*PTPRH*	Protein tyrosine phosphatase, receptor type, H	19q13.42	Membrane	−1.644
*SCN3A*	Sodium channel, voltage-gated, type III, alpha subunit	2q24.3	Drug-target, membrane	−0.443
*SNAP91*	Synaptosomal-associated protein, 91 K-Da homologue (mouse)	6q14.2	Membrane	−0.969
*SOX4*	SRY (sex determining region Y)-box 4	6p22.3	TF, development, transcription regulator activity	−1.651

Coefficients calculated in the training set

*TF* transcriptional factor

^a^Ensembl cytogenetic band

^b^Coefficients of two separate probes for the same gene

### Development and validation of the 18-gene Stage4NB risk score in independent test sets

**Fig. 2:**
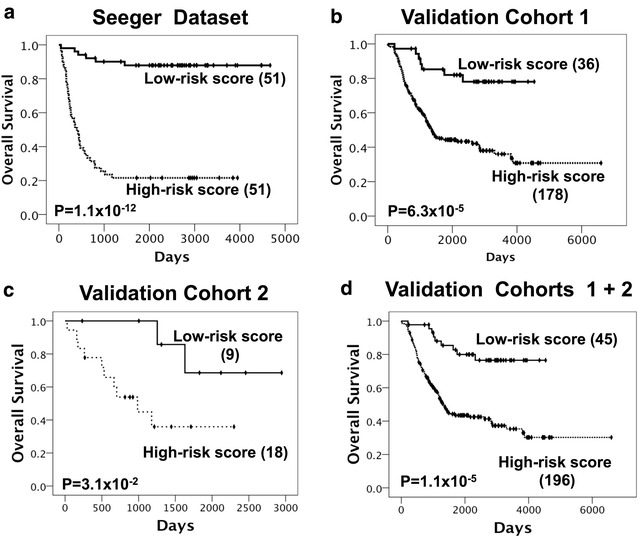
Kaplan–Meier analysis plots of the two subgroups in three independent datasets (**a**–**c**) classified using the 18-gene Stage 4 NB risk score and **d** in combined NB validation cohorts. Number of patients in predicted subgroups is between *brackets*

We generated an outcome predictor of a minimal size and maximum accuracy using 18 unique genes and a model that is based on the relative contributions of each gene. The risk score for each patient was calculated using the regression coefficient of each gene in the 18-gene signature (Table 2). Patients in the Seeger training set were dichotomized according to their 18-gene Stage4NB risk score, and OS was significantly worse in the patient group with a high-risk score (*P* = 1.1 × 10^−12^; Fig. 2a). The 5-year survival in low- and high-risk groups in the Seeger cohort was 88 ± 5 and 20 ± 6 %, respectively. Then, gene expression data from validation cohort 1 were analyzed using the 18-gene Stage4NB risk score. With direct application of the Cox regression coefficient from the Seeger training set and the 50th percentile cutoff threshold, OS in the two patient groups differed significantly in validation cohort 1 (*P* = 6.3 × 10^−5^; Fig. 2b). Finally, the 18-gene Stage4NB risk score was further validated using an independent cohort of 27 tumors profiled by the same array platform (4 × 44 K oligonucleotide microarrays, Agilent). Again, the Stage4NB risk score separated patient subgroups with a more favorable and an unfavorable outcome (*P* = 3.1 × 10^−2^; Fig. 2c). The 5-year OS of patients classified to be favorable or unfavorable was 82 ± 7 and 43 ± 4 % for validation cohort 1, respectively, and 73 ± 16 and 31 ± 12 % for validation cohort 2, respectively. In the pooled validation cohorts, the 18-gene Stage4NB risk score showed high capability in identifying patients at different risk levels (*P* = 1.1 × 10^−5^; 5 years OS: 80 ± 6, 42 ± 4 %; Fig. 2d). The same cutoff threshold predicted very well the patients’ event-free survival in validation cohort 1 whereas its prediction ability was less marked in validation cohort 2 (Additional file 1: Fig. S4). One possible explanation could be that the classifier is more suitable for the identification of patients at different risk levels of death as it has been built by using only OS data which is in line with the purpose of this research work.

### The 18-gene Stage4NB risk score is an independent prognostic factor

**Fig. 3:**
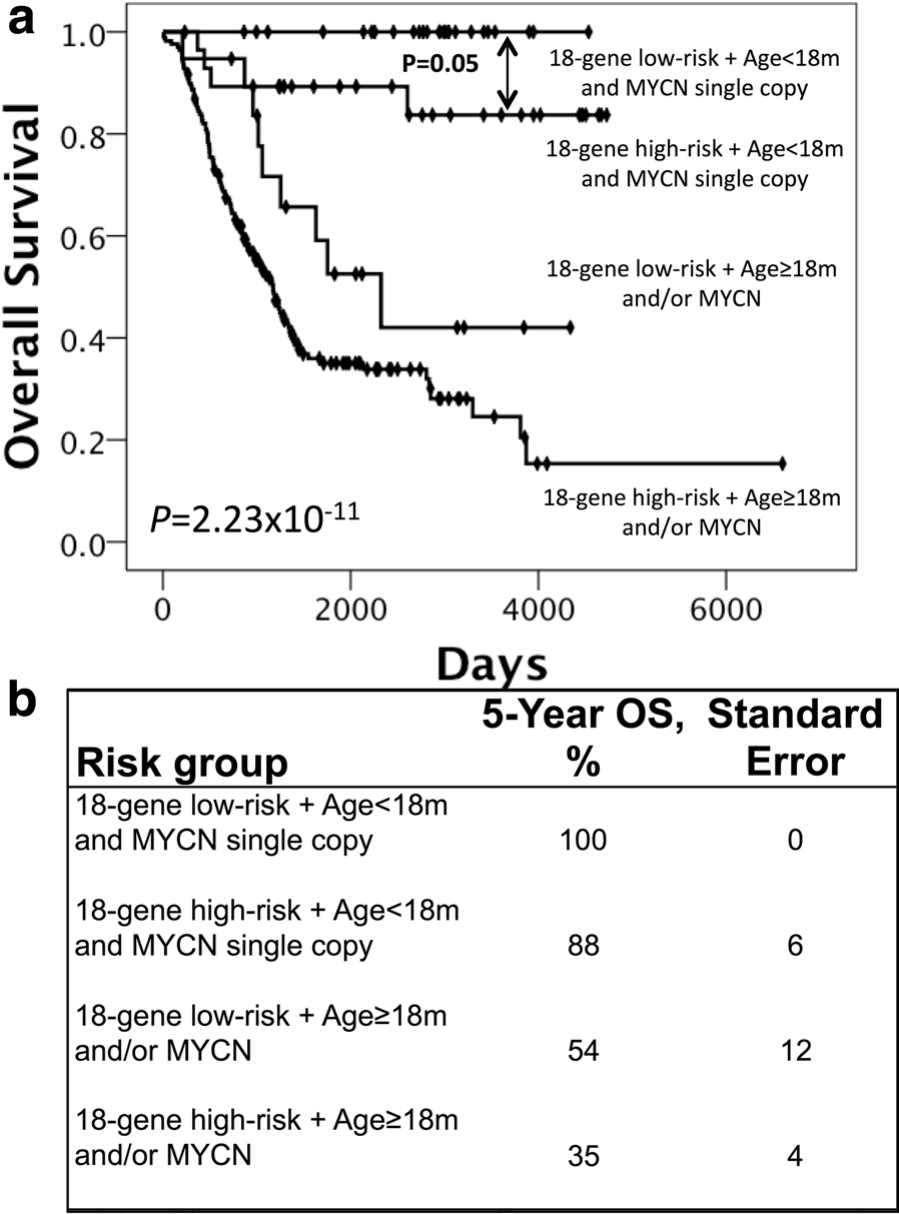
**a** Prognostic impact of the 18-gene Stage4NB risk score, *MYCN* amplification and age ≥18 months. **b** Kaplan–Meier estimates of the rate of survival at 5-years according to combination of the 18-gene Stage4NB risk score, *MYCN* and age

To test whether the risk score is an independent prognostic factor in the pooled validation cohorts, we performed multivariate Cox regression analysis of available prognostic factors, including the risk score, age at diagnosis and *MYCN* status. The risk score turned out to be a significant prognostic marker in addition to age and the *MYCN* status (Table 3). We therefore combined these three prognostic markers in a novel risk stratification system for stage 4 NB. Kaplan–Meier analysis of the pooled validation cohort according to this system showed that age ≥18 months and/or *MYCN* amplification identify subsets with significantly differing survival among favorably and unfavorably classified patients (Fig. 3a). The combination of these three independent prognostic variables thus provided an accurate tool to identify subgroups of patients with a substantially distinct risk to die from disease, ranging from a 5-year survival of 35 ± 4 % to 100 % (*P* = 2.23 × 10^−11^), respectively (Fig. 3b). The 18-gene Stage4NB risk score also showed a high capability in identifying patients at different risk levels in patient groups with age <18 months (P = 0.004), age ≥18 months (*P* = 0.04), age <18 months and *MYCN* non-amplified, and age ≥18 months (*P* = 0.089) and *MYCN* non-amplified (*P* = 0.087) (Additional file 1: Fig. S5). By contrast, our risk score was less accurate in predicting outcome of children with *MYCN*-amplified tumors (*P* = 0.26). In this subgroup, the vast majority of patients (79/81) was classified as high-risk (Additional file 1: Fig. S5). This observation might be due to the fact that the 18-gene risk score consisted mainly of genes involved in neuronal differentiation, which are generally repressed in *MYCN* over-expressing cells [24] (Additional file 1: Table S6; Fig. S6).

**Table 3:** Multivariate Cox regression models for combined NB validation cohorts based on OS considering single prognostic marker and the 18-gene Stage4 NB risk score

Marker	Hazard ratio	95 % CI	*P* value
Validation cohort 1 + 2 (n = 241)			
Age (≥18 vs < 18 months)	2.64	1.579–4.414	0.00020
MYCN (amplified *vs* not amplified)	2.549	1.744–3.727	0.000001
18-gene Stage 4 NB risk score (high vs low risk score groups)	2.237	1.098–4.554	0.027

*OS* overall survival, *N/S* not significant, *NB* neuroblastoma

### Gene network and GO analysis

All 18 genes were down-regulated in the subgroup of patients with a high risk score as compared to those with a low risk score in all analyzed datasets (Additional file 1: Fig. S6). To evaluate the potential functional relevance of the signature genes, we performed gene network and GO analyses. The results showed that “activation of protein kinase A (PKA) activity” was the most enriched biological term (Additional file 1: Fig. S7; Table S6) which is highly involved in neuronal differentiation [25].

## Discussion

By applying a multistep exploration and validation strategy, we identified and validated a risk score-based classifier using the expression patterns of 18 genes that is able to identify two subsets of stage 4 NB patients with different OS. Children with a high risk score uniformly had a poor outcome, with 20–43 % OS at 5 years after diagnosis, whereas those with a low risk score had 73–88 % OS. Moreover, several lines of evidence strongly support that the 18-gene Stage4NB risk score is an independent and significant predictor of prognosis. First, the risk score was a significant predictive factor for OS in multivariate analysis including *MYCN* status and age at diagnosis. Second, the combination of the three independent prognostic variables Stage4NB risk score, age and *MYCN* provided an accurate tool to identify a specific subgroup of patients with favorable outcome (5-year rate of survival of 100 %). Taken together, these results strongly support the notion that the 18-gene risk score identifies groups of patients at different risk levels of death in stage 4 patients and that may represent an accurate tool to improve risk estimation of patients who are currently believed to be at high risk to die from disease.

Recent research has elucidated the biology of NB allowing more accurate stratification, which has permitted to develop appropriate treatments for children with localized tumors reducing cytotoxic therapy and increasing the survival rate [1]. Major challenges still remain for children with metastatic (stage 4) NB older than 18 months or those whose tumors are *MYCN*-amplified, with 5-year survival rates of only 30–40 % [1]. Our findings suggest that the 18-gene Stage4NB risk score may be capable to improve the current risk stratification system of high-risk patients, as it is able to identify a subset of patients with favorable outcome who may require less intensive therapies. The reduction of treatment intensities for patients with a more favorable outcome may substantially decrease the risk of serious side effects such as sepsis, primary hypothyroidism, growth hormone deficiency, deafness and cardiovascular problems. In our cohort, 22 patients classified as low-risk by our risk score had been treated according to a high-risk protocol. The favorable outcome of these patients indicates that they might have had a similar outcome with less intensive treatment. Thus, we propose that reduction of cytotoxic intensities in such patients should be evaluated prospectively.

Our model included genes that may be interesting for further research based on either their chromosomal location, their known function, or their possible role as drug targets (*ADCY1*, *AKR1C1* and *SCNA3*). Not surprisingly, this set of 18 predictive genes contains numerous genes that have been reported to have a role in the neuronal differentiation which if arrested contributes to early event in NB pathogenesis as also demonstrated by our recent work on genetic susceptibility to NB [26]. For instance, *ADCY1GNAI1* and *PRKACB* genes are associated with the cAMP-mediated signaling which plays a crucial role in initiating differentiation in transformed and embryonic cells of neuronal and glial origin [27]. cAMP-stimulating agents also induce differentiation in human and mouse NB cells [27]. *ARHGEF10L* gene is a member of the Rho family of guanine nucleotide exchange factors (GEF) that activate Rho GTPases. Interestingly, frequent mutations of RAC-RHO pathway genes regulating neuritogenesis have been found in NBs stage 3 and 4 [28]. Further genes reported to have a role in neuronal differentiation are *HOXC6* [29], *SOX4* [30], *FOXP1* [31], *GFRA3* [32], and *PTPRH* [33]. Our recent study shows the biological role of *FOXP1* in contributing to NB progression and unfavorable patient outcome [34]. This is in line with the evidence that high risk neuroblastomas are characterized by low expression of genes involved in neuronal differentiation [15, 35–37]. Importantly, the gene network and GO analysis showed that “PKA activity”, which includes *ADCY1* and *PRKACB* genes, was the most enriched biological term. *ADCY1* encodes a form of adenylate cyclase whereas *PRKACB* encodes a catalytic subunit of PKA [27]. Both genes show lower expression values in patients classified to be at high risk. Recently, adenylyl cyclases have emerged as potential drug target in diverse diseases [38] whereas PKA signaling pathway is known to antagonize Hedgehog signaling [39]. Interestingly, the activation of PKA pathway by forskolin (*ADCY1* activator) has been associated with a reduction of cell proliferation and an induction of apoptosis by inhibition of Hedgehog signal in NB cell lines [40]. Moreover, a recent study demonstrated that the neuropeptide pituitary adenylyl cyclase activating polypeptide (PACAP), another *ADCY1* activator, inhibits proliferation of primary medulloblastoma derived tumorsphere cultures by PKA activation and inhibition of Hedgehog signal [41]. Together, these data support the idea that regulation of PKA signaling by *ADCY1* activation might be an additional therapeutic strategy for stage 4 NB. The correlation of high *AKR1C1* expression with cancer is supported by two recently studies [42, 43], possibly due to the ability of *AKR1C1* to act as tumor suppressor gene. Particularly in stromal fibroblasts and carcinoma cells, high *AKR1C1* expression correlates with favorable tumor characteristics and longer survival in primary breast cancer patients [43]. Recent advances in the molecular biology of esophageal squamous cell carcinoma (ESCC) have shown that in ESCC patients, high *AKR1C1* expression increase the sensitive of ESCC cells to ethyl-3,4-dihydroxybenzoate (EDHB) providing potential guidance for the chemoprevention of ESCC [42]. *AKR1C1* shows lower expression values in high risk NB patients and these data supported that therapeutic modulation of the *AKR1C1* expression, could be an attractive therapeutic possibility for patients classified to be at high risk.

In conclusion, we have established and validated a robust prognostic scoring system in the largest stage 4 NB population to date (n = 342). Our study provides further evidence that gene expression-based classification works well in NB. We demonstrated an excellent performance of our classifier on independent data sets, involving stage 4 patients from different countries and using two validation cohorts. The gene-based risk score shows high performance in risk estimation of stage 4 NB patients alone and when integrated with the currently used variables age and *MYCN* status. Together, our findings encourage large prospective studies on the clinical value of the scoring system, which may ultimately improve risk assessment and treatment stratification of children with metastatic NB.

## Conclusions

In the last decade, survival rate of children with advanced stage 4 neuroblastoma, the most common solid extracranial tumor of infancy, has improved little whereas remarkable progresses have been recorded for children with localized or 4s disease. Gene expression–based classification has been demonstrated to precisely predict neuroblastoma outcome; however, no such classifier is used in clinical practice to date. Here we present a risk estimation for children affected by advanced stage 4 neuroblastoma which integrates both a highly accurate gene expression–based classifier and established prognostic markers. According to this system, we identified novel subgroups of patients with favourable prognosis among high-risk patients. Our study may help clinicians in choosing a more appropriate therapy to reduce side effects for those children with low-risk profile or a more intensive treatments for those children with high-risk profile.

## Additional file

10.1186/s12967-016-0896-7 The supplementary document contains detailed information on the methods used, supplementary figures and tables.

## Abbreviations

INSS: international neuroblastoma staging system
NB: neuroblastoma
OS: overall survival
GO: gene ontology
