# Supplementary material for: An 18 gene expression-based score classifier predicts the clinical outcome in stage 4 neuroblastoma
Source: J Transl Med. 2016 May 17;14:142. doi: 10.1186/s12967-016-0896-7 (PMC4870777; doi:10.1186/s12967-016-0896-7)
Supplement: Supplementary file 1 — 10.1186/s12967-016-0896-7 The supplementary document contains detailed information on the methods used, supplementary figures and tables. [file 12967_2016_896_MOESM1_ESM.docx]

**An 18 gene expression-based score classifier predicts the clinical outcome in stage 4 neuroblastoma**

**Supplementary Materials and Methods**

**Gene Selection**

We selected 520 genes/probes with high potential clinical and biological relevance in neuroblastoma (NB). The gene selection method was based on three main points (see also the Figure 1 in the main document).

**First**, we performed a Medline search through the PubMed database (1990-2014) by using the keywords “Neuronal marker” and “Neuroendocrine marker”. One important histological feature of high stage NB is lack of neuronal differentiation which [[1](#_ENREF_1), [2](#_ENREF_2)] has been demonstrate to have prognostic value [[3](#_ENREF_3)]. We thus selected the studies that were in accordance with the following major criteria:

1. Studies of neuroscience and developmental biology describing the cellular and molecular mechanisms by which complex nervous systems emerge;
2. Studies of clinical research investigating the clinicopathological impact of genes in tumor diseases;
3. Studies of drug-induced differentiation describing differential gene expression in cell models treated with neuronal differentiating agents.

These criteria identified a total of 115 articles and 203 genes (Supplementary Table 1 and Supplementary Table 2).

**Second**, diverse microarray studies on global gene expression of NB specimens have been published using different technical platforms and analysis methods. However, the overlap in identified expressed genes with prognostic information between these studies is low. We speculated that using genes derived from diverse accurately designed signatures could potentially add high prognostic value and reproducibility to a new scoring system for stage 4 patients. We therefore selected the following published gene signatures of NB outcome:

a) 112 genes Oberthuer et al., 2006 [[4](#_ENREF_4)]

b) 55 genes Asgharzadeh et al., 2006 [[5](#_ENREF_5)]

c) 59 genes Vermeulen et al., 2009 [[6](#_ENREF_6)]

d) 32 genes Fardin et al., 2010 [[7](#_ENREF_7)]

e) 14 genes Asgharzadeh et al., 2012 [[8](#_ENREF_8)].

The gene names are reported in the Supplementary Table 1 and Supplementary Table 2.

**Third**, morphologic differentiation to ganglioneuromatous histopathology has for decades been recognized as a positive prognostic sign in NB. Retinoic acid (RA) is a potent inducer of differentiation in NB cells. We thus performed a re-analysis of public microarray data obtained from NB cell lines treated with all-trans retinoic acid as described below.

*Analysis of NB cell lines microarray data*

We obtained 81 genes (101 unique probes) differently expressed after treatment with RA of NB cell lines by analyzing microarray data freely available at GEO database (accession number: GSE9169). This microarray dataset contained data of two SH-SY5Y subclones and one SK-N-SH cell lines treated with RA at six time points (0 h, 6 h, 1 day, 2 days, 3 days and 5 days). The microarray data of each cell line were separately normalized using the gcRMA method in the Affy package in the R language environment. SAM (Significance Analysis of Microarrays) method implemented in MeV software (*v*. 4.8) was used to identify genes that significantly differed among the different time points [[9](#_ENREF_9)]. SAM computes a statistic analyses for each gene, measuring the strength of the relationship between gene expression and treatment. We used repeated permutations of the data with default parameters to determine if the expression of each gene was significantly related to RA treatment. The cut-off for significance was determined by a tuning parameter delta-value based on the false positive rate. This analysis identified 23, 17 and 74 genes/probes (13 out of 114 shared among the three gene lists) differentially expressed in SHSY5Y (ATCC), SHSY5Y (ECACC), SK-N-SH cell lines, respectively (Supplementary Table 3). Next, the gene-lists from the three independent experiments were compared to create a list of unique genes (n=101). The Venn diagram in Figure 1a shows that the genes *CYP26A1* and *CYP26B1*, codifying for enzymes involved in metabolism of retinoids, were found in the three gene lists. The gene ontology (GO) identified the biological process “fat-soluble vitamin metabolic process” as the most representative GO term among the all three gene lists (Figure 1b). These data prove the robustness of our microarray analysis.


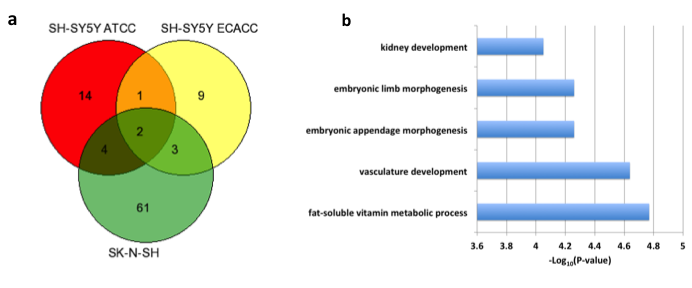


**Figure 1**. (a) Venn diagram of genes obtained by re-analysis of retinoic acid-induced NB cell lines. Two genes (*CYP26A1* and *CYP26B1*) were found in all of the three genes lists. Three genes (*CFI, DTL* and *MPPED2*) were found in two gene lists from SH-SY5Y ECACC and SK-N-SH cell lines. One gene (*C4orf6*) was found in SH-SY5Y ECACC and SH-SY5Y ATCC cell lines. Four genes (*CRABP2, DHRS3, ENPP2, NCAM2*) were found in two gene lists from SK-N-SH and SH-SY5Y ATCC cell lines. (b) Gene ontology (GO) analysis (DAVID website tool) of genes obtained by re-analysis retinoic acid-induced neuroblastoma (NB) cell lines. Only GO terms of biological process with nominal P-values less than 0.0001 are plotted.

**Identification of the optimal gene set to predict overall survival**

We selected these two datasets as both contained all 520 gene/probes identified by the strategy described above. As shown in the Fig. 1 and Supplementary Fig. S1, we computed the Cox regression coefficient-based risk score for each stage 4 NB patient that is based on the relative contributions of each of the 520 gene/probes in the univariate analysis, as described in the following equation:

where xi represents the gene expression obtained by microarray data and αi is referred to the regression coefficient obtained by Cox regression analysis of each single gene/probe. The negative regression coefficient value indicated that higher expression correlated with longer survival while the positive regression coefficient indicated that higher expression correlated with shorter survival. The regression coefficients for each gene/probe are reported in the Supplementary Table S1 and S2. By this first analysis, we produced 520 cox regression coefficient-based risk scores obtained by an incrementally larger set of genes from the 520-probe/gene list ranked by prognostic significance to predict the survival of 102 and 40 stage 4 samples respectively from Seeger and Versteeg dataset (Supplementary Fig. S1). The second step was to identify the best Cox regression coefficient-based risk score able to predict the survival. We thus performed receiver operating characteristic (ROC) curve analysis of Cox regression coefficient-based risk scores of patients. After generating ROC curves, we assessed the performance of the 520 risk scores by comparing the areas under the curve (AUC), where a larger AUC indicates better performance. We selected the optimal predictor composed of 18 genes, which maximized the AUC (Supplementary Fig. S2 and S3).

**Validation datasets and RNA preparation**

In summary, the tumor samples were checked by a pathologist before RNA isolation. Subsequently, samples with at least 60% tumor content were utilized and total RNA was isolated from B50 mg of snap-frozen neuroblastoma tissue obtained before chemotherapeutic treatment. After homogenization of tumor tissue by using the FastPrep FP120 cell disruptor (Qbiogene, Carlsbad, CA, USA) total RNA was isolated using the TRIzol reagent (Invitrogen, Karlsruhe, Germany). Integrity of the isolated RNA was assessed using the 2100 Bioanalyzer (Agilent Technologies) and only samples with an RNA integrity number of at least 7.5 were considered for further processing.

**Microarray technology**

In summary, 1µg total of tumor RNA was linearily amplified and labeled with Cy3 using Agilent’s one-color Quick Amp Labeling Kit following the instructions of the protocol. Then, 1650 ng of Cy3-labeled cRNA was hybridized on the 4x44K arrays (GPL16876) using Agilent’s High-RPM Gene Expression Hyb Kit. Hybridization was performed for 17 h at 65°C in a rotating hyb oven at 10 rpm according to the company’s recommendations. After washing and scanning, resulting TIFF-images were processed using Agilent’s Feature Extraction software Version 9.5.1. Finally, raw data were normalized using the quantile algorithm from limma. Raw data and the matrix of normalized intensity values were submitted to GEO database (GSE79910).

**Gene network and Gene ontology analysis**

We used the website tool GeneMANIA (http://www.genemania.org) to find gene groups that are involved in the same signaling. GeneMANIA finds other genes that are related to a set of input genes, using a very large set of functional interaction data [[10](#_ENREF_10)]. Interaction data include protein and genetic interactions, pathways, co-expression, co-localization and protein domain similarity. We searched the GeneMANIA website using the 18 genes of our predictor underlying specific functional themes to find out how the genes interact with each other and most enriched biological functions associated with the genes. The resulting sub-network containing our query genes and additional related genes helped us to identify the genetic pathways deregulated in patients categorized as high-risk (Supplementary Figure 5).

**The genes of outcome predictor**

The Supplementary Table 4 shows the genes/probes included in the optimal predictor.

No gene derived by re-analysis of RA NB induced cell lines and from 32-gene signature [[7](#_ENREF_7)] that is specific signature including genes involved in hypoxia tumor environment. Interestingly, two genes (*SNAP91*, synaptosomal-associated protein 91kDa and *SCN3A*, sodium channel, voltage-gated, type III, alpha subunit) derived from Medline search [[11](#_ENREF_11), [12](#_ENREF_12)]. *SNAP91* gene encodes clathrin coatassembly protein AP180 which has been implicated in regulating neurite outgrowth [[12](#_ENREF_12)]. *SCN3A* gene encodes one member of the sodium channel alpha subunit gene family, which is responsible for the initiation and propagation of action potentials in the nervous system [[12](#_ENREF_12)]. The 18 genes included in our model showed low expression levels in the patients with high risk score (Supplementary Figure 6).

**References**

1. Benard J, Raguenez G, Kauffmann A, Valent A, Ripoche H, Joulin V, Job B, Danglot G, Cantais S, Robert T, et al: **MYCN-non-amplified metastatic neuroblastoma with good prognosis and spontaneous regression: a molecular portrait of stage 4S.** *Mol Oncol* 2008, **2:**261-271.

2. Fischer M, Oberthuer A, Brors B, Kahlert Y, Skowron M, Voth H, Warnat P, Ernestus K, Hero B, Berthold F: **Differential expression of neuronal genes defines subtypes of disseminated neuroblastoma with favorable and unfavorable outcome.** *Clin Cancer Res* 2006, **12:**5118-5128.

3. Fredlund E, Ringner M, Maris JM, Pahlman S: **High Myc pathway activity and low stage of neuronal differentiation associate with poor outcome in neuroblastoma.** *Proc Natl Acad Sci U S A* 2008, **105:**14094-14099.

4. Oberthuer A, Berthold F, Warnat P, Hero B, Kahlert Y, Spitz R, Ernestus K, Konig R, Haas S, Eils R, et al: **Customized oligonucleotide microarray gene expression-based classification of neuroblastoma patients outperforms current clinical risk stratification.** *J Clin Oncol* 2006, **24:**5070-5078.

5. Asgharzadeh S, Pique-Regi R, Sposto R, Wang H, Yang Y, Shimada H, Matthay K, Buckley J, Ortega A, Seeger RC: **Prognostic significance of gene expression profiles of metastatic neuroblastomas lacking MYCN gene amplification.** *J Natl Cancer Inst* 2006, **98:**1193-1203.

6. Vermeulen J, De Preter K, Naranjo A, Vercruysse L, Van Roy N, Hellemans J, Swerts K, Bravo S, Scaruffi P, Tonini GP, et al: **Predicting outcomes for children with neuroblastoma using a multigene-expression signature: a retrospective SIOPEN/COG/GPOH study.** *Lancet Oncol* 2009, **10:**663-671.

7. Fardin P, Barla A, Mosci S, Rosasco L, Verri A, Versteeg R, Caron HN, Molenaar JJ, Ora I, Eva A, et al: **A biology-driven approach identifies the hypoxia gene signature as a predictor of the outcome of neuroblastoma patients.** *Mol Cancer* 2010, **9:**185.

8. Asgharzadeh S, Salo JA, Ji L, Oberthuer A, Fischer M, Berthold F, Hadjidaniel M, Liu CW, Metelitsa LS, Pique-Regi R, et al: **Clinical significance of tumor-associated inflammatory cells in metastatic neuroblastoma.** *J Clin Oncol* 2012, **30:**3525-3532.

9. Tusher VG, Tibshirani R, Chu G: **Significance analysis of microarrays applied to the ionizing radiation response.** *Proc Natl Acad Sci U S A* 2001, **98:**5116-5121.

10. Warde-Farley D, Donaldson SL, Comes O, Zuberi K, Badrawi R, Chao P, Franz M, Grouios C, Kazi F, Lopes CT, et al: **The GeneMANIA prediction server: biological network integration for gene prioritization and predicting gene function.** *Nucleic Acids Res* 2010, **38:**W214-220.

11. Bushlin I, Petralia RS, Wu F, Harel A, Mughal MR, Mattson MP, Yao PJ: **Clathrin assembly protein AP180 and CALM differentially control axogenesis and dendrite outgrowth in embryonic hippocampal neurons.** *J Neurosci* 2008, **28:**10257-10271.

12. Catterall WA: **Voltage-gated sodium channels at 60: structure, function and pathophysiology.** *J Physiol* 2012, **590:**2577-2589.


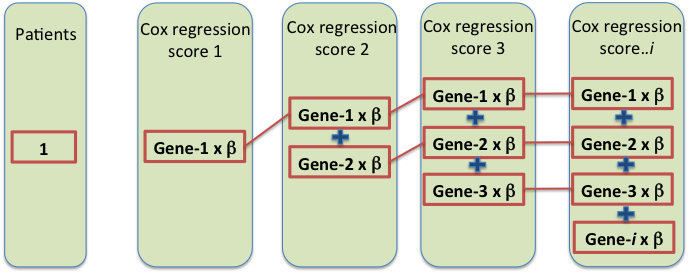


**Supplementary Figure S1.** Method used for computing the Cox regression coefficient-based risk score of stage 4 neuroblastoma (NB) patients. Based on the significance values obtained by Cox regression analysis, the 520 genes have been ranked starting from the gene that better predicted the patient survival (gene-1, gene-2, gene-3…. gene-520). This computation was performed for each patient of both datasets Seeger and Versteeg. The figure shows only one patient as an example. βetha value indicates the cox regression coefficient.


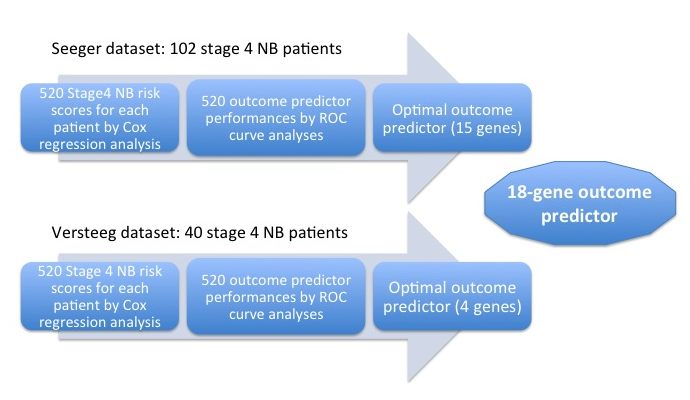


**Supplementary Figure S2.** Overall method used for identifying the predictive genes of stage 4 neuroblastoma (NB) outcome. One gene was found in the two-separated analyses. In the Seeger dataset, two genes were identified by two unique probes (total genes/probes = 20).


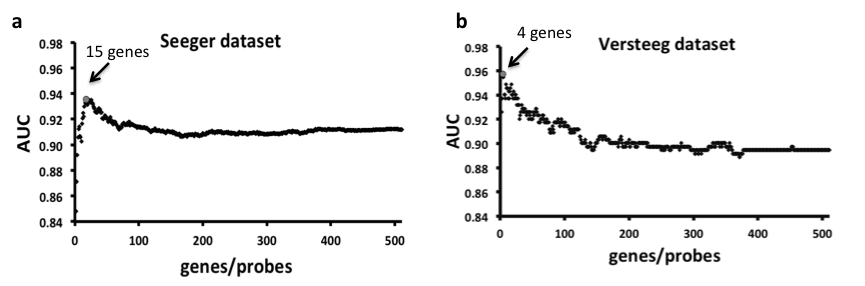


**Supplementary Figure S3.** Graph representing AUC values according to the risk scores obtained by incrementally larger set of genes from the 520-probe/gene list ranked by prognostic significance to predict the survival in (a) Seeger (15 genes) and (b) Versteeg (4 genes) microarray dataset of stage 4 patients. One gene was found in the two-separated analyses.

**Supplementary Figure S4.** Kaplan-Meier analysis plots of the two subgroups in two independent datasets (a-b) classified using the 18-gene Stage4NB risk score and (c) in combined NB validation cohorts. Number of patients in predicted subgroups is between brackets.


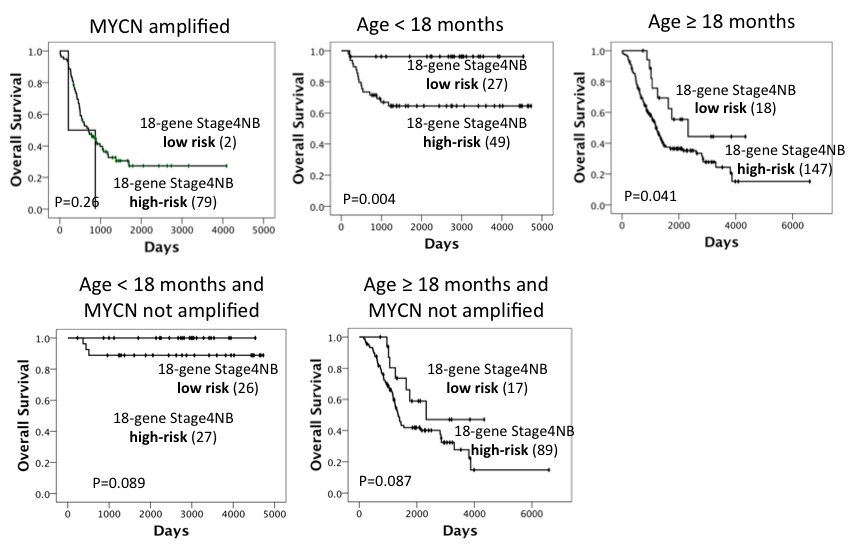


**Supplementary Figure S5.** Kaplan-Meier analysis of stage 4 patients of pooled validation datasets in 5 patient clinical sub-groups stratified according to the 18-gene Stage4NB risk score. Number of patients in predicted sub-groups is between brackets.


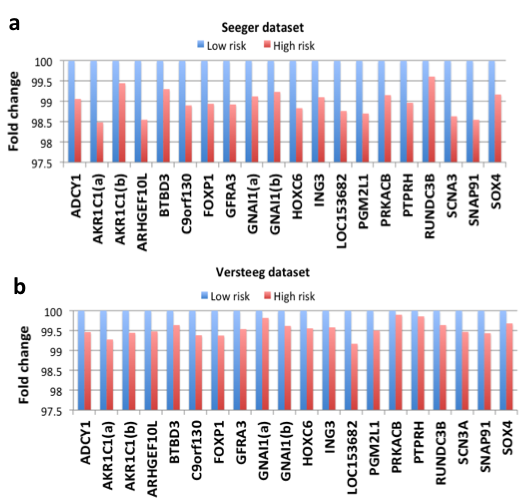


**Supplementary Figure S6.** Expression values of the 18 genes in the sub-group patients with high-risk score with respect to those with low-risk score in (a) Seeger and (b) Versteeg dataset. The same trend was observed in validation cohorts (data not shown).

**Supplementary Figure S7.** Gene network and gene ontology analysis. The black color indicates the queried genes whereas the grey color indicates the predicted interacting genes. The red color indicates the genes included in the most enriched biological theme: “activation of protein kinase A activity”.

| **Supplementary Table 1**. Selected probe/gene (s) with their original source and coefficient regression calculated using gene expression values of Seeger dataset | | | | | | | | |  |
| --- | --- | --- | --- | --- | --- | --- | --- | --- | --- |
| Probe | Gene | Medline Search | 144 gene signature (Oberthuer et al., 2006) | 55 gene signature (Asgharzadeh et al., 2006) | 59 gene signature (Vermeulen et al., 2009) | 32 gene signature (Fardin et al, 2010) | 14 gene signature (Asgharzadeh et al., 2012) | 101 genes from NB cell lines treated with retinoic acid (GSE9169) | Coefficient regression |
| 233139_at | A2BP1 | + |  |  |  |  |  |  | -0.148 |
| 242007_at | AB073353 |  |  | + |  |  |  |  | -0.630 |
| 209735_at | ABCG2 | + |  |  |  |  |  |  | -0.487 |
| 228416_at | ACVR2A | + |  |  |  |  |  |  | -0.605 |
| 213245_at | ADCY1 |  |  | + |  |  |  |  | -0.964 |
| 232062_at | ADCY1 |  |  | + |  |  |  |  | 0.764 |
| 230237_at | ADCYAP1 | + |  |  |  |  |  |  | -0.002 |
| 200903_s_at | AHCY |  | + |  | + |  |  |  | 0.368 |
| 221569_at | AHI1 |  | + |  |  |  |  |  | -1.540 |
| 225342_at | AK3L1 |  |  |  |  | + |  |  | 0.043 |
| 204151_x_at | AKR1C1 |  | + |  | + |  |  |  | -0.682 |
| 216594_x_at | AKR1C1 |  | + |  |  |  |  |  | -0.740 |
| 207163_s_at | AKT1 | + |  |  |  |  |  |  | -0.916 |
| 202022_at | ALDOC |  |  |  |  | + |  |  | -0.695 |
| 222108_at | AMIGO2 |  | + |  |  |  |  |  | -0.364 |
| 223333_s_at | ANGPTL4 |  |  |  |  | + |  | + | 1.051 |
| 227337_at | ANKRD37 |  |  |  |  | + |  |  | 0.896 |
| 209871_s_at | APBA2 |  |  | + |  |  |  |  | -0.902 |
| 214953_s_at | APP | + |  |  |  |  |  |  | -0.483 |
| 210090_at | ARC | + |  |  |  |  |  |  | 0.540 |
| 228368_at | ARHGAP20 |  |  |  |  |  |  | + | 0.589 |
| 221656_s_at | ARHGEF10L |  | + |  |  |  |  |  | -2.903 |
| 235412_at | ARHGEF7 |  |  |  | + |  |  |  | -0.001 |
| 209988_s_at | ASCL1 | + |  |  |  |  |  |  | 0.130 |
| 204608_at | ASL |  |  |  |  |  |  | + | 0.358 |
| 226941_at | ATF6 | + |  |  |  |  |  |  | -1.033 |
| 208079_s_at | AURKA |  | + |  |  |  |  |  | 0.393 |
| 207848_at | AVP | + |  |  |  |  |  |  | 0.696 |
| 206435_at | B4GALNT1 | + |  |  |  |  |  |  | -1.512 |
| 203685_at | BCL2 | + |  |  |  |  |  |  | -0.808 |
| 206382_s_at | BDNF | + |  |  |  |  |  |  | 0.500 |
| 239367_at | BDNF | + |  |  |  |  |  |  | 0.250 |
| 201170_s_at | BHLHB2 |  |  |  |  | + |  |  | 0.233 |
| 202095_s_at | BIRC5 | + |  |  |  |  |  |  | -0.102 |
| 210334_x_at | BIRC5 | + |  |  | + |  |  |  | 0.057 |
| 202094_at | BIRC5 | + |  |  |  |  |  |  | -0.007 |
| 241986_at | BMPER |  |  |  |  |  |  | + | -0.003 |
| 243117_at | BNIP3 |  |  |  |  | + |  |  | 0.106 |
| 221478_at | BNIP3L |  |  |  |  | + |  |  | -1.914 |
| **Supplementary Table 1 continue**. Selected probe/gene (s) with their original source and coefficient regression calculated using gene expression values of Seeger dataset | | | | | | | | |  |
| Probe | Gene | Medline Search | 144 gene signature (Oberthuer et al., 2006) | 55 gene signature (Asgharzadeh et al., 2006) | 59 gene signature (Vermeulen et al., 2009) | 32 gene signature (Fardin et al, 2010) | 14 gene signature (Asgharzadeh et al., 2012) | 101 genes from NB cell lines treated with retinoic acid (GSE9169) | Coefficient regression |
| 221703_at | BRIP1 |  | + |  |  |  |  |  | 0.072 |
| 202946_s_at | BTBD3 |  |  | + |  |  | + |  | -0.936 |
| 200921_s_at | BTG1 |  |  |  |  | + |  |  | -0.196 |
| 225981_at | C17orf28 | + |  |  |  |  |  |  | -0.411 |
| 244593_at | C17orf28 | + |  |  |  |  |  |  | -0.270 |
| 225065_x_at | C17orf45 |  |  | + |  |  |  |  | -0.632 |
| 219063_at | C1orf35 |  |  | + |  |  |  |  | -0.012 |
| 220942_x_at | C3orf28 |  |  |  |  | + |  |  | 0.986 |
| 207241_at | C4orf6 |  |  |  |  |  |  | + | 0.002 |
| 220751_s_at | C5orf4 |  | + |  |  |  |  |  | -0.746 |
| 232067_at | C6orf168 |  |  | + |  |  |  |  | -0.480 |
| 239799_at | C9orf130 |  |  | + |  |  |  |  | -1.315 |
| 209726_at | CA11 |  |  |  |  |  |  | + | 0.048 |
| 211802_x_at | CACNA1G |  |  | + |  |  |  |  | 0.112 |
| 204814_at | CADPS | + |  |  |  |  |  |  | -0.571 |
| 233950_at | CADPS | + |  |  |  |  |  |  | -0.447 |
| 236697_at | CADPS | + |  |  |  |  |  |  | 0.578 |
| 234220_at | CADPS | + |  |  |  |  |  |  | -0.392 |
| 217561_at | CALCA |  |  |  |  |  |  | + | 0.506 |
| 217495_x_at | CALCA |  |  |  |  |  |  | + | -0.741 |
| 214636_at | CALCB |  |  |  |  |  |  | + | 0.225 |
| 34846_at | CAMK2B |  | + |  |  |  |  |  | 0.932 |
| 210244_at | CAMP | + |  |  |  |  |  |  | 1.719 |
| 241285_at | CAMTA1 |  | + | + |  |  |  |  | 1.219 |
| 225693_s_at | CAMTA1 |  | + |  |  |  | + |  | 0.558 |
| 227328_at | CAMTA1 |  | + |  | + |  |  |  | 0.168 |
| 212948_at | CAMTA2 |  |  |  | + |  |  |  | -2.047 |
| 229641_at | CCBE1 |  |  |  |  |  |  | + | -0.446 |
| 228729_at | CCNB1 |  | + |  |  |  |  |  | 0.131 |
| 214710_s_at | CCNB1 |  | + |  |  |  |  |  | 0.109 |
| 232768_at | CCNB2 |  | + |  |  |  |  |  | -0.107 |
| 201743_at | CD14 |  |  |  |  |  | + |  | -0.056 |
| 219669_at | CD177 |  |  |  |  |  |  | + | 0.085 |
| 206120_at | CD33 |  |  |  |  |  | + |  | -0.023 |
| 209543_s_at | CD34 | + |  |  |  |  |  |  | 0.705 |
| 204490_s_at | CD44 | + |  |  |  |  |  |  | -0.337 |
| 209835_x_at | CD44 | + |  |  |  |  |  |  | -0.376 |
| 212014_x_at | CD44 | + |  |  |  |  |  |  | -0.376 |
| 212063_at | CD44 | + |  |  |  |  |  |  | -0.243 |
| **Supplementary Table 1 continue**. Selected probe/gene (s) with their original source and coefficient regression calculated using gene expression values of Seeger dataset | | | | | | | | |  |
| Probe | Gene | Medline Search | 144 gene signature (Oberthuer et al., 2006) | 55 gene signature (Asgharzadeh et al., 2006) | 59 gene signature (Vermeulen et al., 2009) | 32 gene signature (Fardin et al, 2010) | 14 gene signature (Asgharzadeh et al., 2012) | 101 genes from NB cell lines treated with retinoic acid (GSE9169) | Coefficient regression |
| 216062_at | CD44 | + |  |  |  |  |  |  | 0.296 |
| 210916_s_at | CD44 | + |  |  | + |  |  |  | -0.758 |
| 204489_s_at | CD44 | + |  |  |  |  |  |  | 0.214 |
| 217523_at | CD44 | + |  |  |  |  |  |  | -0.177 |
| 234411_x_at | CD44 | + |  |  |  |  |  |  | -0.127 |
| 234418_x_at | CD44 | + |  |  |  |  |  |  | -0.068 |
| 216056_at | CD44 | + |  |  |  |  |  |  | 0.139 |
| 201926_s_at | CD55 |  |  |  |  |  |  | + | 0.215 |
| 201925_s_at | CD55 |  |  |  |  |  |  | + | 0.060 |
| 207176_s_at | CD80 | + |  |  |  |  |  |  | 1.208 |
| 201029_s_at | CD99 | + |  |  |  |  |  |  | 0.788 |
| 231534_at | CDC2 |  | + |  |  |  |  |  | 0.226 |
| 203968_s_at | CDC6 |  | + |  |  |  |  |  | -0.333 |
| 224753_at | CDCA5 |  | + |  | + |  |  |  | 0.101 |
| 203440_at | CDH2 | + |  |  |  |  |  |  | -0.506 |
| 203441_s_at | CDH2 | + |  |  |  |  |  |  | -0.299 |
| 237305_at | CDH2 | + |  |  |  |  |  |  | -0.148 |
| 214803_at | CDH6 |  | + |  |  |  |  |  | -1.067 |
| 207039_at | CDKN2A |  |  |  |  |  |  | + | -0.097 |
| 209714_s_at | CDKN3 |  | + |  | + |  |  |  | 0.355 |
| 210821_x_at | CENPA |  | + |  |  |  |  |  | 1.796 |
| 207828_s_at | CENPF |  | + |  |  |  |  |  | 0.085 |
| 207331_at | CENPF |  | + |  |  |  |  |  | 0.025 |
| 228559_at | CENPN |  | + |  |  |  |  |  | 0.481 |
| 218542_at | CEP55 |  | + |  |  |  |  |  | 0.103 |
| 203854_at | CFI |  |  |  |  |  |  | + | 0.506 |
| 225817_at | CGNL1 |  | + |  |  |  |  |  | -0.105 |
| 229808_at | CHAF1A | + | + |  |  |  |  |  | 1.733 |
| 203976_s_at | CHAF1A | + |  |  |  |  |  |  | -0.130 |
| 203975_s_at | CHAF1A | + |  |  |  |  |  |  | 0.049 |
| 214426_x_at | CHAF1A | + |  |  |  |  |  |  | -0.046 |
| 215727_x_at | CHD3 |  |  | + |  |  |  |  | 0.129 |
| 217250_s_at | CHD5 |  |  |  | + |  |  |  | -0.328 |
| 204697_s_at | CHGA | + |  |  |  |  |  |  | -0.026 |
| 210123_s_at | CHRNA7 | + |  |  |  |  |  |  | -0.418 |
| 204170_s_at | CKS2 |  | + |  |  |  |  |  | 0.355 |
| 225759_x_at | CLMN | + |  |  |  |  |  |  | -0.335 |
| 201561_s_at | CLSTN1 |  |  |  | + |  |  |  | -0.832 |
| 204375_at | CLSTN3 | + |  |  |  |  |  |  | -0.889 |
| **Supplementary Table 1 continue**. Selected probe/gene (s) with their original source and coefficient regression calculated using gene expression values of Seeger dataset | | | | | | | | |  |
| Probe | Gene | Medline Search | 144 gene signature (Oberthuer et al., 2006) | 55 gene signature (Asgharzadeh et al., 2006) | 59 gene signature (Vermeulen et al., 2009) | 32 gene signature (Fardin et al, 2010) | 14 gene signature (Asgharzadeh et al., 2012) | 101 genes from NB cell lines treated with retinoic acid (GSE9169) | Coefficient regression |
| 205518_s_at | CMAH |  |  |  |  |  |  | + | 0.554 |
| 228437_at | CNIH4 |  | + |  |  |  |  |  | -0.048 |
| 208243_s_at | CNR1 | + | + | + |  |  |  |  | -1.443 |
| 203477_at | COL15A1 |  |  |  |  |  |  | + | 0.210 |
| 202404_s_at | COL1A2 |  |  |  |  |  |  | + | -0.084 |
| 201438_at | COL6A3 |  |  |  |  |  |  | + | 0.044 |
| 221019_s_at | COLEC12 |  |  |  |  |  |  | + | 1.758 |
| 202575_at | CRABP2 |  |  |  |  |  |  | + | 0.356 |
| 205143_at | CSPG3 |  |  |  | + |  |  |  | 0.223 |
| 209151_x_at | CTNND2 |  | + |  |  |  |  |  | 0.019 |
| 204533_at | CXCL10 | + |  |  |  |  |  |  | 0.073 |
| 209687_at | CXCL12 | + |  |  |  |  |  |  | 0.253 |
| 217028_at | CXCR4 | + |  |  |  |  |  |  | 0.088 |
| 206504_at | CYP24A1 |  |  |  |  |  |  | + | 0.845 |
| 206424_at | CYP26A1 |  |  |  |  |  |  | + | 0.505 |
| 219825_at | CYP26B1 |  |  |  |  |  |  | + | 0.550 |
| 216646_at | DCC1 |  | + |  |  |  |  |  | -0.561 |
| 204850_s_at | DCX | + |  |  |  |  |  |  | -0.662 |
| 236775_s_at | DDC |  |  |  | + |  |  |  | -0.274 |
| 212690_at | DDHD2 |  |  | + |  |  |  |  | -0.990 |
| 209383_at | DDIT3 | + |  |  |  |  |  |  | 0.303 |
| 202887_s_at | DDIT4 |  |  |  |  | + |  |  | 0.554 |
| 235545_at | DEPDC1 |  |  |  |  |  |  | + | -0.477 |
| 238032_at | DHRS3 |  |  |  |  |  |  | + | 0.982 |
| 202481_at | DHRS3 |  |  |  |  |  |  | + | 0.799 |
| 213661_at | DKFZP586H2123 | | + |  |  |  |  |  | 0.133 |
| 203764_at | DLG7 |  | + |  |  |  |  |  | 0.170 |
| 210227_at | DLGAP2 |  |  | + |  |  |  |  | 0.672 |
| 209560_s_at | DLK1 |  |  |  |  |  |  | + | 0.078 |
| 208382_s_at | DMC1 | + |  |  |  |  |  |  | 0.840 |
| 208386_x_at | DMC1 | + |  |  |  |  |  |  | -0.178 |
| 239059_at | DNAH1 |  |  | + |  |  |  |  | 0.302 |
| 226281_at | DNER |  | + |  |  |  |  |  | -0.285 |
| 201430_s_at | DPYSL3 |  |  |  | + |  |  |  | -0.872 |
| 215810_x_at | DST | + | + |  |  |  |  |  | 0.098 |
| 218585_s_at | DTL |  |  |  |  |  |  | + | -0.129 |
| 222680_s_at | DTL |  | + |  |  |  |  | + | 0.016 |
| 228033_at | E2F7 |  |  |  |  |  |  | + | 0.113 |
| 233261_at | EBF1 |  | + |  |  |  |  |  | 0.691 |
| **Supplementary Table 1 continue**. Selected probe/gene (s) with their original source and coefficient regression calculated using gene expression values of Seeger dataset | | | | | | | | |  |
| Probe | Gene | Medline Search | 144 gene signature (Oberthuer et al., 2006) | 55 gene signature (Asgharzadeh et al., 2006) | 59 gene signature (Vermeulen et al., 2009) | 32 gene signature (Fardin et al, 2010) | 14 gene signature (Asgharzadeh et al., 2012) | 101 genes from NB cell lines treated with retinoic acid (GSE9169) | Coefficient regression |
| 244876_at | EBF1 |  | + |  |  |  |  |  | -0.097 |
| 219914_at | ECEL1 | + |  |  | + |  |  |  | -0.629 |
| 221497_x_at | EGLN1 |  |  |  |  | + |  |  | 0.465 |
| 219232_s_at | EGLN3 |  |  |  |  | + |  |  | -0.042 |
| 201303_at | EIF4A3 | + |  |  |  |  |  |  | -1.003 |
| 234904_x_at | ELAVL4 |  |  |  | + |  |  |  | -0.889 |
| 210839_s_at | ENPP2 |  |  |  |  |  |  | + | -0.253 |
| 209392_at | ENPP2 |  |  |  |  |  |  | + | -0.230 |
| 200878_at | EPAS1 | + |  |  |  |  |  |  | 0.003 |
| 206710_s_at | EPB41L3 |  |  |  | + |  |  |  | -0.166 |
| 227449_at | EPHA4 |  |  |  |  |  |  | + | -0.316 |
| 231239_at | EPHA5 |  |  |  | + |  |  |  | -0.309 |
| 203463_s_at | EPN2 |  |  |  | + |  |  |  | -1.672 |
| 235588_at | ESCO2 |  | + |  |  |  |  |  | 0.439 |
| 209214_s_at | EWSR1 | + |  |  |  |  |  |  | -1.122 |
| 204603_at | EXO1 |  | + |  |  |  |  |  | 0.566 |
| 204363_at | F3 | + |  |  |  |  |  |  | 0.353 |
| 225572_at | FAM119A | + |  |  |  |  |  |  | -0.946 |
| 219895_at | FAM70A |  | + |  |  |  |  |  | -0.435 |
| 225834_at | FAM72A |  | + |  |  |  |  |  | 0.074 |
| 235948_at | FAM80A | + |  |  |  |  |  |  | 0.917 |
| 225687_at | FAM83D |  | + |  |  |  |  |  | 0.103 |
| 211623_s_at | FBL |  | + |  |  |  |  |  | -0.252 |
| 202766_s_at | FBN1 |  |  |  |  |  |  | + | 0.314 |
| 204006_s_at | FCGR3A |  |  |  |  |  | + |  | 0.163 |
| 237707_at | FLJ20105 |  | + |  |  |  |  |  | -0.311 |
| 229280_s_at | FLJ22536 |  | + |  |  |  |  |  | -1.036 |
| 228281_at | FLJ25416 |  |  |  |  |  |  | + | 0.828 |
| 239203_at | FLJ39575 |  | + |  |  |  |  |  | -1.618 |
| 209189_at | FOS | + |  |  |  |  |  |  | 0.202 |
| 229844_at | FOXP1 |  |  | + |  |  |  |  | -0.984 |
| 230645_at | FRMD3 |  |  |  |  |  |  | + | -0.410 |
| 204948_s_at | FST |  |  |  |  |  |  | + | 0.739 |
| 243037_at | FUBP1 |  |  | + |  |  |  |  | 0.055 |
| 238551_at | FUT11 |  |  |  |  | + |  |  | 0.259 |
| 243006_at | FYN |  |  |  | + |  |  |  | -0.402 |
| 208869_s_at | GABARAPL1 |  | + |  |  |  |  |  | -0.748 |
| 204471_at | GAP43 | + |  |  |  |  |  | + | -0.874 |
| 216963_s_at | GAP43 | + |  |  |  |  |  |  | -0.753 |
| **Supplementary Table 1 continue**. Selected probe/gene (s) with their original source and coefficient regression calculated using gene expression values of Seeger dataset | | | | | | | | |  |
| Probe | Gene | Medline Search | 144 gene signature (Oberthuer et al., 2006) | 55 gene signature (Asgharzadeh et al., 2006) | 59 gene signature (Vermeulen et al., 2009) | 32 gene signature (Fardin et al, 2010) | 14 gene signature (Asgharzadeh et al., 2012) | 101 genes from NB cell lines treated with retinoic acid (GSE9169) | Coefficient regression |
| 216967_at | GAP43 | + |  |  |  |  |  |  | -0.143 |
| 238076_at | GATAD2B |  |  | + |  |  |  |  | -0.441 |
| 221577_x_at | GDF15 |  |  |  |  |  |  | + | 0.760 |
| 203540_at | GFAP | + |  |  |  |  |  |  | 0.621 |
| 229259_at | GFAP | + |  |  |  |  |  |  | 0.074 |
| 214479_at | GFRA3 |  |  | + |  |  | + |  | -0.916 |
| 214431_at | GMPS |  | + |  |  |  |  |  | 1.027 |
| 227692_at | GNAI1 |  | + | + |  |  |  |  | -1.049 |
| 209576_at | GNAI1 |  |  | + |  |  |  |  | -1.484 |
| 241865_at | GNB1 |  |  |  | + |  |  |  | -0.230 |
| 224634_at | GPATCH4 |  |  |  |  |  | + |  | -0.118 |
| 219898_at | GPR85 |  |  | + |  |  |  |  | -1.055 |
| 203632_s_at | GPRC5B |  |  |  |  |  |  | + | 0.455 |
| 218468_s_at | GREM1 |  |  |  |  |  |  | + | -0.166 |
| 218469_at | GREM1 |  |  |  |  |  |  | + | -0.040 |
| 210411_s_at | GRIN2B | + |  |  |  |  |  |  | 0.140 |
| 227085_at | H2AFV |  |  | + |  |  | + |  | -0.478 |
| 211222_s_at | HAP1 |  |  | + |  |  |  |  | 0.905 |
| 237295_at | HECW1 | + |  |  |  |  |  |  | 0.646 |
| 210331_at | HECW1 | + |  |  |  |  |  |  | -0.732 |
| 243743_at | HECW1 | + |  |  |  |  |  |  | -0.418 |
| 215584_at | HECW1 | + |  |  |  |  |  |  | -0.004 |
| 232080_at | HECW2 |  |  |  |  |  |  | + | -0.644 |
| 242890_at | HELLS |  |  |  |  |  |  | + | -0.155 |
| 200989_at | HIF1A | + |  |  |  |  |  |  | 0.751 |
| 209398_at | HIST1H1C |  | + |  |  |  |  |  | 0.634 |
| 212641_at | HIVEP2 |  | + |  | + |  |  |  | -1.343 |
| 220042_x_at | HIVEP3 |  |  | + |  |  |  |  | 0.135 |
| 216548_x_at | HMG4L |  | + |  |  |  |  |  | 0.485 |
| 243368_at | HMGB2 |  | + |  |  |  |  |  | 0.448 |
| 206858_s_at | HOXC6 |  |  | + |  |  |  |  | -0.786 |
| 231936_at | HOXC9 | + | + |  |  |  |  |  | -0.627 |
| 231906_at | HOXD8 |  |  |  |  |  |  | + | -0.501 |
| 206864_s_at | HRK |  |  | + |  |  |  |  | -0.387 |
| 235892_at | Hs.125166 |  |  |  |  |  |  | + | 0.117 |
| 243023_at | Hs.27996 |  |  | + |  |  |  |  | -0.340 |
| 227061_at | Hs.86538 |  |  |  |  |  |  | + | 0.237 |
| 229714_at | HS6ST3 |  | + |  |  |  |  |  | -0.193 |
| 206638_at | HTR2B |  |  |  |  |  |  | + | 0.382 |
| **Supplementary Table 1 continue**. Selected probe/gene (s) with their original source and coefficient regression calculated using gene expression values of Seeger dataset | | | | | | | | |  |
| Probe | Gene | Medline Search | 144 gene signature (Oberthuer et al., 2006) | 55 gene signature (Asgharzadeh et al., 2006) | 59 gene signature (Vermeulen et al., 2009) | 32 gene signature (Fardin et al, 2010) | 14 gene signature (Asgharzadeh et al., 2012) | 101 genes from NB cell lines treated with retinoic acid (GSE9169) | Coefficient regression |
| 230249_at | ICK |  |  | + |  |  |  |  | -0.220 |
| 226757_at | IFIT2 |  |  |  |  |  |  | + | 0.029 |
| 203627_at | IGF1R |  |  |  |  | + |  |  | -0.294 |
| 212143_s_at | IGFBP3 |  |  |  |  | + |  |  | 0.321 |
| 228375_at | IGSF11 |  |  |  |  |  |  | + | 0.981 |
| 232793_at | IGSF4 |  |  |  | + |  |  |  | -0.077 |
| 226333_at | IL-6R |  |  |  |  |  | + |  | -0.141 |
| 207433_at | IL10 |  |  |  |  |  | + |  | 0.435 |
| 211506_s_at | IL8 |  |  |  |  |  |  | + | 0.313 |
| 230719_at | ING3 |  |  | + |  |  |  |  | -0.918 |
| 202794_at | INPP1 |  |  |  | + |  |  |  | -1.210 |
| 240841_at | INSM2 |  |  |  |  |  |  | + | -0.434 |
| 222240_s_at | ISYNA1 |  |  |  |  |  |  | + | -0.077 |
| 213416_at | ITGA4 |  |  |  |  |  |  | + | 0.236 |
| 212689_s_at | JMJD1A |  |  |  |  | + |  |  | -1.108 |
| 212805_at | KIAA0367 | + |  |  |  |  |  |  | -0.437 |
| 212806_at | KIAA0367 | + |  |  |  |  |  |  | -0.587 |
| 213839_at | KIAA0500 |  |  |  |  |  |  | + | -0.613 |
| 236641_at | KIF14 |  |  |  |  |  |  | + | -0.002 |
| 226968_at | KIF1B |  | + |  |  |  |  |  | -0.834 |
| 205051_s_at | KIT | + |  |  |  |  |  |  | 0.081 |
| 205266_at | LIF | + |  |  |  |  |  |  | 0.584 |
| 204424_s_at | LMO3 | + |  |  |  |  |  |  | 1.238 |
| 231348_s_at | LMO3 | + |  |  |  |  |  |  | -0.314 |
| 217378_x_at | LOC100130100 | |  | + |  |  |  |  | 0.369 |
| 228338_at | LOC120376 |  |  |  |  |  |  | + | 0.058 |
| 232794_at | LOC153682 |  |  | + |  |  |  |  | -0.886 |
| 214162_at | LOC284244 |  |  | + |  |  |  |  | -0.617 |
| 230117_at | LOC285878 |  | + |  |  |  |  |  | -0.386 |
| 228880_at | LOC339984 | + |  |  |  |  |  |  | -0.077 |
| 228564_at | LOC375295 |  |  |  |  |  |  | + | -0.051 |
| 222150_s_at | LOC54103 |  |  |  |  |  |  | + | 0.192 |
| 200785_s_at | LRP1 | + |  |  |  |  |  |  | 0.453 |
| 209841_s_at | LRRN3 | + |  |  |  |  |  |  | -0.677 |
| 209840_s_at | LRRN3 | + |  |  |  |  |  |  | -0.233 |
| 203362_s_at | MAD2L1 |  | + |  |  |  |  |  | 0.037 |
| 224650_at | MAL2 |  |  |  |  |  |  | + | 0.223 |
| 203266_s_at | MAP2K4 |  |  |  | + |  |  |  | -1.324 |
| 202890_at | MAP7 |  | + |  |  |  |  |  | -0.328 |
| **Supplementary Table 1 continue**. Selected probe/gene (s) with their original source and coefficient regression calculated using gene expression values of Seeger dataset | | | | | | | | |  |
| Probe | Gene | Medline Search | 144 gene signature (Oberthuer et al., 2006) | 55 gene signature (Asgharzadeh et al., 2006) | 59 gene signature (Vermeulen et al., 2009) | 32 gene signature (Fardin et al, 2010) | 14 gene signature (Asgharzadeh et al., 2012) | 101 genes from NB cell lines treated with retinoic acid (GSE9169) | Coefficient regression |
| 215471_s_at | MAP7 | + | + |  | + |  |  |  | -0.830 |
| 202889_x_at | MAP7 |  | + |  |  |  |  |  | -0.791 |
| 203930_s_at | MAPT |  | + |  | + | + |  |  | -0.658 |
| 209072_at | MBP | + |  |  |  |  |  |  | 0.941 |
| 201620_at | MBTPS1 | + |  |  |  |  |  |  | -0.835 |
| 220651_s_at | MCM10 |  |  |  |  |  |  | + | -0.010 |
| 202107_s_at | MCM2 |  |  |  | + |  |  |  | -0.136 |
| 238977_at | MCM6 |  | + |  |  |  |  |  | -0.280 |
| 201930_at | MCM6 |  | + |  |  |  |  |  | -0.174 |
| 209035_at | MDK |  |  |  |  |  |  | + | -0.058 |
| 242172_at | MEIS1 | + |  |  |  |  |  |  | -0.310 |
| 202645_s_at | MEN1 |  | + |  |  |  |  |  | 0.499 |
| 223728_at | MGC16385 |  |  | + |  |  |  |  | -0.058 |
| 229305_at | MLF1IP |  | + |  |  |  |  |  | -0.306 |
| 204475_at | MMP1 |  |  |  |  |  |  | + | -0.307 |
| 203878_s_at | MMP11 |  |  |  |  |  |  | + | -0.179 |
| 223700_at | MND1 |  | + |  |  |  |  |  | 0.046 |
| 205413_at | MPPED2 |  |  |  |  |  |  | + | -0.202 |
| 208787_at | MRPL3 |  | + |  | + |  |  |  | 0.695 |
| 223276_at | MST150 |  |  |  |  |  |  | + | 0.629 |
| 223172_s_at | MTP18 |  |  |  |  | + |  |  | 1.406 |
| 203036_s_at | MTSS1 |  |  |  | + |  |  |  | -0.106 |
| 202364_at | MXI1 |  |  |  |  | + |  |  | -1.153 |
| 209757_s_at | MYCN | + |  |  |  |  |  |  | -0.858 |
| 209756_s_at | MYCN | + |  |  |  |  |  |  | -0.616 |
| 242026_at | MYCN | + |  |  | + |  |  |  | 1.446 |
| 234376_at | MYCN | + |  |  |  |  |  |  | 0.683 |
| 211377_x_at | MYCN | + |  |  |  |  |  |  | -0.744 |
| 232227_at | NALP1 |  | + |  |  |  |  |  | 0.264 |
| 229799_s_at | NCAM1 | + |  |  |  |  |  |  | 0.409 |
| 232390_at | NCAM2 |  |  |  |  |  |  | + | -0.098 |
| 205669_at | NCAM2 |  |  |  |  |  |  | + | -0.370 |
| 240991_at | NDRG1 |  |  |  |  | + |  |  | 0.095 |
| 33767_at | NEFH | + |  |  |  |  |  |  | 0.142 |
| 221916_at | NEFL | + |  |  |  |  |  |  | -0.136 |
| 211080_s_at | NEK2 |  | + |  |  |  |  |  | 0.085 |
| 206282_at | NEUROD1 | + |  |  |  |  |  |  | 0.336 |
| 210162_s_at | NFATC1 | + |  |  |  |  |  |  | 0.789 |
| 201146_at | NFE2L2 | + |  |  |  |  |  |  | 0.319 |
| **Supplementary Table 1 continue**. Selected probe/gene (s) with their original source and coefficient regression calculated using gene expression values of Seeger dataset | | | | | | | | |  |
| Probe | Gene | Medline Search | 144 gene signature (Oberthuer et al., 2006) | 55 gene signature (Asgharzadeh et al., 2006) | 59 gene signature (Vermeulen et al., 2009) | 32 gene signature (Fardin et al, 2010) | 14 gene signature (Asgharzadeh et al., 2012) | 101 genes from NB cell lines treated with retinoic acid (GSE9169) | Coefficient regression |
| 205858_at | NGFR | + |  |  |  |  |  |  | 0.311 |
| 215228_at | NHLH2 | + |  |  | + |  |  |  | 0.391 |
| 214497_s_at | NHLH2 | + |  |  |  |  |  |  | 0.493 |
| 205004_at | NKRF | + |  |  |  |  |  |  | -1.431 |
| 226797_at | NME1 | + |  |  |  |  |  |  | -0.747 |
| 201577_at | NME1 | + |  |  | + |  |  |  | 0.141 |
| 201268_at | NME2 | + |  |  |  |  |  |  | 0.562 |
| 231798_at | NOG |  |  |  |  |  |  | + | 0.293 |
| 219110_at | NOLA1 |  | + |  |  |  |  |  | 1.292 |
| 206001_at | NPY | + |  |  |  |  |  | + | -0.075 |
| 204105_s_at | NRCAM |  | + |  | + |  |  |  | -0.595 |
| 208605_s_at | NTRK1 | + | + | + | + |  |  |  | -0.357 |
| 214680_at | NTRK2 | + |  | + |  |  | + |  | 0.376 |
| 221796_at | NTRK2 |  |  |  |  |  |  | + | 0.151 |
| 236095_at | NTRK2 |  |  |  |  |  |  | + | 0.084 |
| 229463_at | NTRK2 |  |  |  |  |  |  | + | 0.032 |
| 215115_x_at | NTRK3 | + |  |  |  |  |  |  | -0.493 |
| 217377_x_at | NTRK3 | + |  |  |  |  |  |  | 0.585 |
| 215025_at | NTRK3 | + |  |  |  |  |  |  | -0.324 |
| 217033_x_at | NTRK3 | + |  |  |  |  |  |  | 0.341 |
| 228849_at | NTRK3 | + |  |  |  |  |  |  | -0.007 |
| 206462_s_at | NTRK3 | + |  |  |  |  |  |  | 0.000 |
| 223381_at | NUF2 |  | + |  |  |  |  |  | 0.207 |
| 232377_at | NXPH1 |  | + |  |  |  |  |  | -0.558 |
| 200790_at | ODC1 |  |  |  | + |  |  |  | 0.024 |
| 202733_at | P4HA2 |  |  |  |  | + |  |  | 0.019 |
| 214664_at | PAICS |  | + |  | + |  |  |  | 1.494 |
| 202954_at | PAK3 |  | + |  |  |  |  |  | 0.014 |
| 219148_at | PBK |  | + |  |  |  |  |  | -0.033 |
| 212148_at | PBX1 |  |  |  |  |  |  | + | -0.903 |
| 212151_at | PBX1 |  |  |  |  |  |  | + | -1.051 |
| 208620_at | PCBP1 | + |  |  |  |  |  |  | -0.755 |
| 209361_s_at | PCBP4 | + |  |  |  |  |  |  | -0.697 |
| 201202_at | PCNA |  | + |  |  |  |  |  | -0.167 |
| 212390_at | PDE4DIP |  | + |  | + |  |  |  | -0.670 |
| 210305_at | PDE4DIP |  | + |  |  |  |  |  | -0.307 |
| 206686_at | PDK1 |  |  |  |  | + |  |  | -0.200 |
| 210825_s_at | PEBP1 | + |  |  |  |  |  |  | -1.013 |
| 214660_at | PELO |  |  |  |  |  |  | + | 0.584 |
| **Supplementary Table 1 continue**. Selected probe/gene (s) with their original source and coefficient regression calculated using gene expression values of Seeger dataset | | | | | | | | |  |
| Probe | Gene | Medline Search | 144 gene signature (Oberthuer et al., 2006) | 55 gene signature (Asgharzadeh et al., 2006) | 59 gene signature (Vermeulen et al., 2009) | 32 gene signature (Fardin et al, 2010) | 14 gene signature (Asgharzadeh et al., 2012) | 101 genes from NB cell lines treated with retinoic acid (GSE9169) | Coefficient regression |
| 226731_at | PELO |  |  |  |  |  |  | + | 0.072 |
| 228499_at | PFKFB4 |  |  |  |  | + |  |  | -0.381 |
| 200738_s_at | PGK1 |  |  |  |  | + |  |  | 0.320 |
| 201968_s_at | PGM1 |  |  |  |  | + |  |  | 0.077 |
| 229256_at | PGM2L1 |  | + |  |  |  |  |  | -1.075 |
| 238417_at | PGM2L1 |  | + | + |  |  | + |  | -1.131 |
| 207009_at | PHOX2B | + |  |  |  |  |  |  | -0.463 |
| 212249_at | PIK3R1 |  |  |  | + |  |  |  | -0.509 |
| 231120_x_at | PKIB |  | + |  |  |  |  |  | -0.145 |
| 209318_x_at | PLAGL1 |  |  |  | + |  |  |  | -0.069 |
| 201860_s_at | PLAT |  |  |  | + |  |  | + | 0.139 |
| 200827_at | PLOD1 |  |  |  |  | + |  |  | 0.274 |
| 206471_s_at | PLXNC1 |  | + |  |  |  |  |  | -1.494 |
| 210139_s_at | PMP22 |  | + |  | + |  |  |  | -0.673 |
| 203422_at | POLD1 |  | + |  |  |  |  |  | 0.390 |
| 208510_s_at | PPARG |  |  |  |  |  |  | + | 0.746 |
| 204566_at | PPM1D | + |  |  |  |  |  |  | -0.593 |
| 204086_at | PRAME | + |  |  | + |  |  |  | 0.225 |
| 205277_at | PRDM2 |  |  |  | + |  |  |  | -2.021 |
| 201923_at | PRDX4 |  | + |  |  |  |  |  | 0.228 |
| 211743_s_at | PRG2 |  |  | + |  |  |  |  | -0.175 |
| 226069_at | PRICKLE1 |  |  |  |  |  |  | + | -0.480 |
| 202742_s_at | PRKACB |  | + |  | + |  |  |  | -0.821 |
| 220846_s_at | PRKCZ |  |  |  | + |  |  |  | 0.953 |
| 204304_s_at | PROM1 | + |  |  |  |  |  |  | 0.762 |
| 227192_at | PRRT2 | + |  |  |  |  |  |  | -0.409 |
| 205515_at | PRSS12 |  |  |  |  |  |  | + | 0.455 |
| 213802_at | PRSS12 |  |  |  |  |  |  | + | 0.228 |
| 223536_at | PSD2 |  | + |  |  |  |  |  | -0.832 |
| 209465_x_at | PTN | + |  |  |  |  |  |  | -0.157 |
| 211737_x_at | PTN | + |  |  |  |  |  |  | -0.193 |
| 209466_x_at | PTN | + | + |  | + |  |  |  | -0.175 |
| 208408_at | PTN | + |  |  |  |  |  |  | -0.048 |
| 233471_at | PTPN5 |  |  |  |  |  | + |  | 0.251 |
| 212588_at | PTPRC | + |  |  |  |  |  |  | 0.162 |
| 200635_s_at | PTPRF |  |  |  | + |  |  |  | -0.568 |
| 208300_at | PTPRH |  | + |  | + |  |  |  | -1.644 |
| 215740_at | PTPRN2 |  |  |  | + |  |  |  | -0.466 |
| 203554_x_at | PTTG1 |  | + |  |  |  |  |  | 0.110 |
| **Supplementary Table 1 continue**. Selected probe/gene (s) with their original source and coefficient regression calculated using gene expression values of Seeger dataset | | | | | | | | |  |
| Probe | Gene | Medline Search | 144 gene signature (Oberthuer et al., 2006) | 55 gene signature (Asgharzadeh et al., 2006) | 59 gene signature (Vermeulen et al., 2009) | 32 gene signature (Fardin et al, 2010) | 14 gene signature (Asgharzadeh et al., 2012) | 101 genes from NB cell lines treated with retinoic acid (GSE9169) | Coefficient regression |
| 205174_s_at | QPCT |  |  |  | + |  |  |  | -0.430 |
| 209089_at | RAB5A | + |  |  |  |  |  |  | -0.843 |
| 211961_s_at | RAB7 | + |  |  |  |  |  |  | -0.745 |
| 221915_s_at | RANBP1 |  | + |  |  |  |  |  | 2.028 |
| 205080_at | RARB |  |  |  |  |  |  | + | 0.462 |
| 208530_s_at | RARB |  |  |  |  |  |  | + | -0.278 |
| 206392_s_at | RARRES1 |  |  |  |  |  |  | + | 0.397 |
| 201394_s_at | RBM5 | + |  |  |  |  |  |  | -1.272 |
| 204365_s_at | REEP1 |  | + |  |  |  |  |  | -0.666 |
| 204023_at | RFC4 |  | + |  |  |  |  |  | -0.095 |
| 210258_at | RGS13 |  |  |  |  |  |  | + | 0.099 |
| 206518_s_at | RGS9 |  | + |  |  |  |  |  | -0.405 |
| 208924_at | RNF11 |  | + |  |  |  |  |  | -1.139 |
| 213194_at | ROBO1 | + |  |  |  |  |  |  | -0.194 |
| 240558_at | ROBO1 | + |  |  |  |  |  |  | 0.002 |
| 238047_at | RP13-102H20.1 | | + |  |  |  |  |  | -0.076 |
| 215321_at | RPIB9 |  | + |  |  |  |  |  | -1.016 |
| 225541_at | RPL22L1 |  | + |  |  |  |  |  | 0.491 |
| 201406_at | RPL36A |  | + |  |  |  |  |  | 1.103 |
| 201476_s_at | RRM1 |  | + |  |  |  |  |  | -0.055 |
| 209773_s_at | RRM2 |  | + |  |  |  |  |  | -0.063 |
| 209686_at | S100B | + |  |  |  |  |  |  | 0.103 |
| 204035_at | SCG2 |  | + |  | + |  |  |  | -0.477 |
| 208578_at | SCN10A | + |  |  |  |  |  |  | 0.992 |
| 220791_x_at | SCN11A | + |  |  |  |  |  |  | 0.732 |
| 224029_x_at | SCN11A | + |  |  |  |  |  |  | 0.231 |
| 210853_at | SCN11A | + |  |  |  |  |  |  | 0.052 |
| 210383_at | SCN1A | + |  |  |  |  |  |  | -0.230 |
| 206381_at | SCN2A2 | + |  |  |  |  |  |  | -0.515 |
| 210432_s_at | SCN3A | + |  |  |  |  |  |  | -0.443 |
| 232512_at | SCN3A | + |  | + |  |  |  |  | -0.352 |
| 204722_at | SCN3B |  |  | + |  |  |  |  | -0.654 |
| 206981_at | SCN4A | + |  |  |  |  |  |  | 0.923 |
| 207413_s_at | SCN5A | + |  |  |  |  |  |  | 0.125 |
| 239984_at | SCN7A | + | + |  |  |  |  |  | -0.434 |
| 207864_at | SCN7A | + |  |  |  |  |  |  | -0.198 |
| 207049_at | SCN8A | + |  |  |  |  |  |  | 0.049 |
| 206950_at | SCN9A | + |  |  |  |  |  |  | -0.485 |
| 222986_s_at | SCOTIN | + |  |  |  |  |  |  | -0.220 |
| **Supplementary Table 1 continue**. Selected probe/gene (s) with their original source and coefficient regression calculated using gene expression values of Seeger dataset | | | | | | | | |  |
| Probe | Gene | Medline Search | 144 gene signature (Oberthuer et al., 2006) | 55 gene signature (Asgharzadeh et al., 2006) | 59 gene signature (Vermeulen et al., 2009) | 32 gene signature (Fardin et al, 2010) | 14 gene signature (Asgharzadeh et al., 2012) | 101 genes from NB cell lines treated with retinoic acid (GSE9169) | Coefficient regression |
| 215998_at | SDK1 |  |  | + |  |  |  |  | 0.665 |
| 213151_s_at | SEPT7 | + |  |  |  |  |  |  | -1.488 |
| 244508_at | SEPT7 | + |  |  |  |  |  |  | 0.153 |
| 224762_at | SERINC2 |  | + |  |  |  |  |  | -0.144 |
| 211429_s_at | SERPINA1 |  |  |  |  |  |  | + | 0.320 |
| 202833_s_at | SERPINA1 |  |  |  |  |  |  | + | 0.147 |
| 202376_at | SERPINA3 |  |  |  |  |  |  | + | 0.139 |
| 212268_at | SERPINB1 |  |  |  |  |  |  | + | 0.808 |
| 200657_at | SLC25A5 |  | + |  | + |  |  |  | 0.555 |
| 222088_s_at | SLC2A3 |  |  |  |  | + |  |  | 0.167 |
| 224596_at | SLC44A1 | + |  |  |  |  |  |  | -0.290 |
| 202219_at | SLC6A8 |  |  |  | + |  |  |  | -0.205 |
| 227367_at | SLCO3A1 |  | + |  |  |  |  |  | -0.714 |
| 211989_at | SMARCE1 |  |  | + |  |  |  |  | -0.978 |
| 204953_at | SNAP91 | + |  |  |  |  |  |  | -0.969 |
| 205443_at | SNAPC1 |  |  |  | + |  |  |  | 0.874 |
| 219511_s_at | SNCAIP |  |  |  |  |  |  | + | -0.100 |
| 231112_at | SNRPE |  | + |  |  |  |  |  | 0.072 |
| 209842_at | SOX10 | + |  |  |  |  |  |  | 0.220 |
| 201417_at | SOX4 |  |  | + |  |  |  |  | -1.651 |
| 213665_at | SOX4 |  |  | + |  |  |  |  | 0.018 |
| 209891_at | SPBC25 |  | + |  |  |  |  |  | 0.033 |
| 224144_at | SPTBN4 |  |  | + |  |  |  |  | -0.258 |
| 202817_s_at | SS18 | + |  |  |  |  |  |  | -0.405 |
| 200957_s_at | SSRP1 |  | + |  |  |  |  |  | 0.293 |
| 208482_at | SSTR1 | + |  |  |  |  |  |  | -1.244 |
| 235591_at | SSTR1 | + |  |  |  |  |  |  | 0.017 |
| 239031_at | SSTR2 | + |  |  |  |  |  |  | -0.143 |
| 214491_at | SSTR3 | + |  |  |  |  |  |  | -0.252 |
| 214556_at | SSTR4 | + |  |  |  |  |  |  | 0.676 |
| 214555_at | SSTR5 | + |  |  |  |  |  |  | -0.507 |
| 207524_at | ST7 |  |  | + |  |  |  |  | 2.677 |
| 215362_at | ST8SIA2 |  |  | + |  |  |  |  | 0.391 |
| 202694_at | STK17A |  | + |  |  |  |  |  | 0.219 |
| 212112_s_at | STX12 |  | + |  |  |  |  |  | -0.700 |
| 206322_at | SYN3 | + | + |  |  |  |  |  | 0.022 |
| 213200_at | SYP | + |  |  |  |  |  |  | -0.480 |
| 209938_at | TADA2L |  |  | + |  |  |  |  | -0.119 |
| 211052_s_at | TBCD | + |  |  |  |  |  |  | -0.850 |
| **Supplementary Table 1 continue**. Selected probe/gene (s) with their original source and coefficient regression calculated using gene expression values of Seeger dataset | | | | | | | | |  |
| Probe | Gene | Medline Search | 144 gene signature (Oberthuer et al., 2006) | 55 gene signature (Asgharzadeh et al., 2006) | 59 gene signature (Vermeulen et al., 2009) | 32 gene signature (Fardin et al, 2010) | 14 gene signature (Asgharzadeh et al., 2012) | 101 genes from NB cell lines treated with retinoic acid (GSE9169) | Coefficient regression |
| 201174_s_at | TERF2IP | + |  |  |  |  |  |  | -1.066 |
| 207199_at | TERT | + |  |  |  |  |  |  | 1.150 |
| 202720_at | TES |  |  |  |  |  |  | + | 1.015 |
| 203400_s_at | TF | + |  |  |  |  |  |  | -0.198 |
| 220109_at | TF | + |  |  |  |  |  |  | 0.061 |
| 214063_s_at | TF | + |  |  |  |  |  |  | 0.019 |
| 214064_at | TF | + |  |  |  |  |  |  | 0.040 |
| 215686_x_at | TFAP2B | + |  |  |  |  |  |  | 0.093 |
| 208691_at | TFRC | + |  |  |  |  |  |  | 0.476 |
| 208291_s_at | TH | + |  |  |  |  |  |  | -0.223 |
| 230380_at | THAP2 |  |  | + |  |  | + |  | 0.322 |
| 203887_s_at | THBD |  |  |  |  |  |  | + | 0.301 |
| 243103_at | TK1 |  | + |  |  |  |  |  | -0.434 |
| 223557_s_at | TMEFF2 |  |  |  |  |  |  | + | -0.146 |
| 218930_s_at | TMEM106B |  | + |  |  |  |  |  | -0.221 |
| 218834_s_at | TMEM132A | + |  |  |  |  |  |  | -0.417 |
| 219701_at | TMOD2 |  |  | + |  |  |  |  | -0.324 |
| 207113_s_at | TNF | + |  |  |  |  |  |  | 0.580 |
| 211282_x_at | TNFRSF25 |  |  |  | + |  |  |  | -1.030 |
| 207196_s_at | TNIP1 |  |  |  |  | + |  |  | -0.195 |
| 226198_at | TOM1L2 |  | + |  |  |  |  |  | -1.409 |
| 232546_at | TP73 | + |  |  |  |  |  |  | 0.902 |
| 220804_s_at | TP73 | + |  |  |  |  |  |  | 0.465 |
| 213011_s_at | TPI1 |  |  |  |  | + |  |  | -0.328 |
| 202154_x_at | TUBB3 | + |  |  |  |  |  |  | -1.315 |
| 213476_x_at | TUBB3 | + |  |  |  |  |  |  | -1.227 |
| 201009_s_at | TXNIP |  |  |  |  | + |  |  | -0.033 |
| 243016_at | TYMS |  | + |  | + |  |  |  | -0.153 |
| 201387_s_at | UCHL1 | + |  |  |  |  |  |  | -0.779 |
| 204063_s_at | ULK2 |  |  |  | + |  |  |  | -1.049 |
| 231325_at | UNC5D |  |  |  |  |  |  | + | -0.344 |
| 240751_at | USP8 |  |  | + |  |  |  |  | -0.264 |
| 210512_s_at | VEGFA | + |  |  |  |  |  |  | -0.638 |
| 211527_x_at | VEGFA |  |  |  |  | + |  |  | -0.116 |
| 201426_s_at | VIM | + |  |  |  |  |  |  | 0.277 |
| 211598_x_at | VIPR2 | + |  |  |  |  |  |  | 0.390 |
| 203856_at | VRK1 |  | + |  |  |  |  |  | 0.005 |
| 218851_s_at | WDR33 |  |  | + |  |  |  |  | -1.143 |
| 201294_s_at | WSB1 |  | + |  | + |  |  |  | -0.985 |
| **Supplementary Table 1 continue**. Selected probe/gene (s) with their original source and coefficient regression calculated using gene expression values of Seeger dataset | | | | | | | | |  |
| Probe | Gene | Medline Search | 144 gene signature (Oberthuer et al., 2006) | 55 gene signature (Asgharzadeh et al., 2006) | 59 gene signature (Vermeulen et al., 2009) | 32 gene signature (Fardin et al, 2010) | 14 gene signature (Asgharzadeh et al., 2012) | 101 genes from NB cell lines treated with retinoic acid (GSE9169) | Coefficient regression |
| 210561_s_at | WSB1 |  | + |  |  |  |  |  | -0.530 |
| 237802_at | XKR4 |  | + |  |  |  |  |  | -0.448 |
| 227047_x_at | ZBTB4 |  | + |  |  |  |  |  | -0.086 |
| 229551_x_at | ZNF367 |  |  |  |  |  |  | + | -0.092 |
| 222536_s_at | ZNF395 |  |  |  |  | + |  |  | -0.020 |
| 214761_at | ZNF423 | + |  |  |  |  |  |  | -0.463 |
| 217237_at | ZNF423 | + |  |  |  |  |  |  | 0.073 |
| 243567_at | ZNF423 | + |  |  |  |  |  |  | 0.065 |
| 232396_at | ZNF710 |  |  | + |  |  |  |  | 0.632 |
| 206059_at | ZNF91 |  |  | + |  |  |  |  | -0.495 |

| **Supplementary Table 2**. Selected probe/gene (s) with their original source and coefficient regression calculated using the gene expression values of Versteeg dataset | | | | | | | | | |
| --- | --- | --- | --- | --- | --- | --- | --- | --- | --- |
| Probe | Gene | Medline Search | 144 gene signature (Oberthuer et al., 2006) | 55 gene signature (Asgharzadeh et al., 2006) | 59 gene signature (Vermeulen et al., 2009) | 32 gene signature (Fardin et al, 2010) | 14 gene signature (Asgharzadeh et al., 2012) | 101 genes from NB cell lines treated with retinoic acid (GSE9169) | Coefficient regression |
|  |  |  |  |  |  |  |  |  |  |
| 233139_at | A2BP1 | + |  |  |  |  |  |  | -0.124 |
| 242007_at | AB073353 |  |  | + |  |  |  |  | -0.769 |
| 209735_at | ABCG2 | + |  |  |  |  |  |  | -0.232 |
| 228416_at | ACVR2A | + |  |  |  |  |  |  | -0.499 |
| 217237_at | ADAM3B | + |  |  |  |  |  |  | 0.232 |
| 232062_at | ADCY1 |  |  | + |  |  |  |  | -0.144 |
| 213245_at | ADCY1 |  |  | + |  |  |  |  | -0.137 |
| 230237_at | ADCYAP1 | + |  |  |  |  |  |  | -0.065 |
| 215362_at | AF007143 |  |  | + |  |  |  |  | -0.093 |
| 215998_at | AF052150 |  |  | + |  |  |  |  | -0.034 |
| 200903_s_at | AHCY |  | + |  | + |  |  |  | 0.756 |
| 221569_at | AHI1 |  | + |  |  |  |  |  | -0.593 |
| 240558_at | AI023335 | + |  |  |  |  |  |  | -0.006 |
| 239984_at | AI333640 | + |  |  |  |  |  |  | -0.275 |
| 242890_at | AK091544 |  |  |  |  |  |  | + | 0.176 |
| 216594_x_at | AKR1C1 |  | + |  |  |  |  |  | -0.728 |
| 204151_x_at | AKR1C1 |  | + |  | + |  |  |  | -0.655 |
| 207163_s_at | AKT1 | + |  |  |  |  |  |  | -0.523 |
| 202022_at | ALDOC |  |  |  |  | + |  |  | -0.150 |
| 222108_at | AMIGO2 |  | + |  |  |  |  |  | -0.010 |
| 223333_s_at | ANGPTL4 |  |  |  |  |  |  | + | 0.114 |
| 221009_s_at | ANGPTL4 |  |  |  |  | + |  |  | 0.099 |
| 227337_at | ANKRD37 |  |  |  |  | + |  |  | 0.283 |
| 209871_s_at | APBA2 |  |  | + |  |  |  |  | -0.403 |
| 214953_s_at | APP | + |  |  |  |  |  |  | -0.157 |
| 210090_at | ARC | + |  |  |  |  |  |  | 0.267 |
| 228368_at | ARHGAP20 |  |  |  |  |  |  | + | -0.057 |
| 221656_s_at | ARHGEF10L |  | + |  |  |  |  |  | -0.734 |
| 229642_at | ARHGEF7 |  |  |  | + |  |  |  | -0.857 |
| 209988_s_at | ASCL1 | + |  |  |  |  |  |  | 0.188 |
| 204608_at | ASL |  |  |  |  |  |  | + | 0.172 |
| 226941_at | ATF6 | + |  |  |  |  |  |  | 0.429 |
| 204092_s_at | AURKA |  | + |  |  |  |  |  | 0.752 |
| 207848_at | AVP | + |  |  |  |  |  |  | 0.052 |
| 206435_at | B4GALNT1 | + |  |  |  |  |  |  | -0.698 |
| 203685_at | BCL2 | + |  |  |  |  |  |  | -0.241 |
| 206382_s_at | BDNF | + |  |  |  |  |  |  | -0.136 |
| 239367_at | BDNF | + |  |  |  |  |  |  | -0.168 |
| **Supplementary Table 2 continue**. Selected probe/gene (s) with their original source and coefficient regression calculated using the gene expression values of Versteeg dataset | | | | | | | | | |
| Probe | Gene | Medline Search | 144 gene signature (Oberthuer et al., 2006) | 55 gene signature (Asgharzadeh et al., 2006) | 59 gene signature (Vermeulen et al., 2009) | 32 gene signature (Fardin et al, 2010) | 14 gene signature (Asgharzadeh et al., 2012) | 101 genes from NB cell lines treated with retinoic acid (GSE9169) | Coefficient regression |
| 241285_at | BE219324 |  |  | + |  |  |  |  | 0.310 |
| 201170_s_at | BHLHE40 |  |  |  |  | + |  |  | 0.205 |
| 202095_s_at | BIRC5 | + |  |  | + |  |  |  | 0.431 |
| 202094_at | BIRC5 | + |  |  |  |  |  |  | 0.227 |
| 210334_x_at | BIRC5 | + |  |  |  |  |  |  | -0.005 |
| 241986_at | BMPER |  |  |  |  |  |  | + | 0.036 |
| 201849_at | BNIP3 |  |  |  |  | + |  |  | -0.128 |
| 221478_at | BNIP3L |  |  |  |  | + |  |  | -0.173 |
| 221703_at | BRIP1 |  | + |  |  |  |  |  | 0.067 |
| 202946_s_at | BTBD3 |  |  | + |  |  | + |  | -0.224 |
| 200920_s_at | BTG1 |  |  |  |  | + |  |  | 0.256 |
| 228281_at | C11orf82 |  |  |  |  |  |  | + | 0.183 |
| 228338_at | C11orf93 |  |  |  |  |  |  | + | 0.235 |
| 225065_x_at | C17orf76-AS1,SNORD49A,SNORD49B,SNORD65 | | | + |  |  |  |  | -0.050 |
| 219063_at | C1orf35 |  |  | + |  |  |  |  | -0.062 |
| 207241_at | C4orf6 |  |  |  |  |  |  | + | -0.092 |
| 48031_r_at | C5orf4 |  | + |  |  |  |  |  | -0.307 |
| 209726_at | CA11 |  |  |  |  |  |  | + | 0.153 |
| 211802_x_at | CACNA1G |  |  | + |  |  |  |  | 0.181 |
| 209031_at | CADM1 |  |  |  | + |  |  |  | -0.746 |
| 233950_at | CADPS | + |  |  |  |  |  |  | -0.324 |
| 204814_at | CADPS | + |  |  |  |  |  |  | -0.218 |
| 234220_at | CADPS | + |  |  |  |  |  |  | -0.122 |
| 217561_at | CALCA |  |  |  |  |  |  | + | 0.329 |
| 217495_x_at | CALCA |  |  |  |  |  |  | + | 0.219 |
| 214636_at | CALCB |  |  |  |  |  |  | + | 0.204 |
| 209956_s_at | CAMK2B |  | + |  |  |  |  |  | -0.345 |
| 210244_at | CAMP | + |  |  |  |  |  |  | 0.044 |
| 1555370_a_at | CAMTA1 |  | + |  | + |  | + |  | -0.458 |
| 213268_at | CAMTA1 |  | + |  |  |  |  |  | -0.391 |
| 225692_at | CAMTA1 |  | + |  |  |  |  |  | 0.499 |
| 212948_at | CAMTA2 |  |  |  | + |  |  |  | 0.346 |
| 229641_at | CCBE1 |  |  |  |  |  |  | + | -0.130 |
| 228729_at | CCNB1 |  | + |  |  |  |  |  | 0.577 |
| 214710_s_at | CCNB1 |  | + |  |  |  |  |  | 0.571 |
|  |  |  |  |  |  |  |  |  |  |
| **Supplementary Table 2 continue**. Selected probe/gene (s) with their original source and coefficient regression calculated using the gene expression values of Versteeg dataset | | | | | | | | | |
| Probe | Gene | Medline Search | 144 gene signature (Oberthuer et al., 2006) | 55 gene signature (Asgharzadeh et al., 2006) | 59 gene signature (Vermeulen et al., 2009) | 32 gene signature (Fardin et al, 2010) | 14 gene signature (Asgharzadeh et al., 2012) | 101 genes from NB cell lines treated with retinoic acid (GSE9169) | Coefficient regression |
| 202705_at | CCNB2 |  | + |  |  |  |  |  | 0.432 |
| 201743_at | CD14 |  |  |  |  |  | + |  | -0.058 |
| 219669_at | CD177 |  |  |  |  |  |  | + | 0.227 |
| 206120_at | CD33 |  |  |  |  |  | + |  | 0.096 |
| 209543_s_at | CD34 | + |  |  |  |  |  |  | 0.301 |
| 204489_s_at | CD44 | + |  |  |  |  |  |  | -0.205 |
| 217523_at | CD44 | + |  |  |  |  |  |  | -0.182 |
| 212063_at | CD44 | + |  |  | + |  |  |  | -0.155 |
| 212014_x_at | CD44 | + |  |  |  |  |  |  | -0.168 |
| 204490_s_at | CD44 | + |  |  |  |  |  |  | -0.180 |
| 234411_x_at | CD44 | + |  |  |  |  |  |  | -0.162 |
| 209835_x_at | CD44 | + |  |  |  |  |  |  | -0.130 |
| 210916_s_at | CD44 | + |  |  |  |  |  |  | -0.118 |
| 216056_at | CD44 | + |  |  |  |  |  |  | -0.103 |
| 234418_x_at | CD44 | + |  |  |  |  |  |  | -0.155 |
| 1555950_a_at | CD55 |  |  |  |  |  |  | + | 0.085 |
| 201925_s_at | CD55 |  |  |  |  |  |  | + | 0.075 |
| 201926_s_at | CD55 |  |  |  |  |  |  | + | 0.079 |
| 207176_s_at | CD80 | + |  |  |  |  |  |  | -0.122 |
| 201029_s_at | CD99 | + |  |  |  |  |  |  | 0.172 |
| 203967_at | CDC6 |  | + |  |  |  |  |  | -0.030 |
| 224753_at | CDCA5 |  | + |  | + |  |  |  | 0.485 |
| 203440_at | CDH2 | + |  |  |  |  |  |  | 0.514 |
| 203441_s_at | CDH2 | + |  |  |  |  |  |  | 0.196 |
| 207039_at | CDKN2A |  |  |  |  |  |  | + | 0.091 |
| 1555758_a_at | CDKN3 |  | + |  | + |  |  |  | 0.368 |
| 204962_s_at | CENPA,SLC35F6 | | + |  |  |  |  |  | 0.504 |
| 223728_at | CENPBD1 |  |  | + |  |  |  |  | -0.506 |
| 207828_s_at | CENPF |  | + |  |  |  |  |  | 0.472 |
| 209172_s_at | CENPF |  | + |  |  |  |  |  | 0.391 |
| 219555_s_at | CENPN |  | + |  |  |  |  |  | 0.182 |
| 218542_at | CEP55 |  | + |  |  |  |  |  | 0.417 |
| 1555564_a_at | CFI |  |  |  |  |  |  | + | 0.103 |
| 203854_at | CFI |  |  |  |  |  |  | + | 0.099 |
| 225817_at | CGNL1 |  | + |  |  |  |  |  | -0.045 |
| 203976_s_at | CHAF1A | + |  |  |  |  |  |  | 0.397 |
| 214426_x_at | CHAF1A | + | + |  |  |  |  |  | 0.500 |
|  |  |  |  |  |  |  |  |  |  |
| **Supplementary Table 2 continue**. Selected probe/gene (s) with their original source and coefficient regression calculated using the gene expression values of Versteeg dataset | | | | | | | | | |
| Probe | Gene | Medline Search | 144 gene signature (Oberthuer et al., 2006) | 55 gene signature (Asgharzadeh et al., 2006) | 59 gene signature (Vermeulen et al., 2009) | 32 gene signature (Fardin et al, 2010) | 14 gene signature (Asgharzadeh et al., 2012) | 101 genes from NB cell lines treated with retinoic acid (GSE9169) | Coefficient regression |
| 203975_s_at | CHAF1A | + |  |  |  |  |  |  | 0.284 |
| 229808_at | CHAF1A | + |  |  |  |  |  |  | 0.021 |
| 213965_s_at | CHD5 |  |  |  | + |  |  |  | -0.154 |
| 204697_s_at | CHGA | + |  |  |  |  |  |  | 0.174 |
| 210123_s_at | CHRNA7,CHRFAM7A | + |  |  |  |  |  |  | -0.279 |
| 204170_s_at | CKS2 |  | + |  |  |  |  |  | 0.499 |
| 213839_at | CLMN | + |  |  |  |  |  | + | -0.226 |
| 225759_x_at | CLMN | + |  |  |  |  |  |  | -0.209 |
| 201561_s_at | CLSTN1 |  |  |  | + |  |  |  | -0.179 |
| 204375_at | CLSTN3 | + |  |  |  |  |  |  | -0.291 |
| 205518_s_at | CMAHP |  |  |  |  |  |  | + | 0.012 |
| 223993_s_at | CNIH4 |  | + |  |  |  |  |  | 1.042 |
| 213436_at | CNR1 | + | + |  |  |  |  |  | -0.150 |
| 208243_s_at | CNR1 | + |  | + |  |  |  |  | -0.154 |
| 203477_at | COL15A1 |  |  |  |  |  |  | + | 0.170 |
| 202404_s_at | COL1A2 |  |  |  |  |  |  | + | -0.013 |
| 201438_at | COL6A3 |  |  |  |  |  |  | + | 0.077 |
| 221019_s_at | COLEC12 |  |  |  |  |  |  | + | 0.173 |
| 202575_at | CRABP2 |  |  |  |  |  |  | + | 0.131 |
| 225572_at | CREB1 | + |  |  |  |  |  |  | -0.537 |
| 209617_s_at | CTNND2 |  | + |  |  |  |  |  | -0.253 |
| 204533_at | CXCL10 | + |  |  |  |  |  |  | -0.108 |
| 209687_at | CXCL12 | + |  |  |  |  |  |  | 0.080 |
| 217028_at | CXCR4 | + |  |  |  |  |  |  | 0.368 |
| 206504_at | CYP24A1 |  |  |  |  |  |  | + | 0.136 |
| 206424_at | CYP26A1 |  |  |  |  |  |  | + | 0.239 |
| 219825_at | CYP26B1 |  |  |  |  |  |  | + | 0.194 |
| 204850_s_at | DCX | + |  |  |  |  |  |  | -0.574 |
| 205311_at | DDC |  |  |  | + |  |  |  | 0.025 |
| 212690_at | DDHD2 |  |  | + |  |  |  |  | -0.649 |
| 209383_at | DDIT3 | + |  |  |  |  |  |  | -0.273 |
| 202887_s_at | DDIT4 |  |  |  |  | + |  |  | 0.167 |
| 235545_at | DEPDC1 |  |  |  |  |  |  | + | 0.122 |
| 202481_at | DHRS3 |  |  |  |  |  |  | + | 0.174 |
| 210227_at | DLGAP2 |  |  | + |  |  |  |  | 0.343 |
| 209560_s_at | DLK1 |  |  |  |  |  |  | + | 0.145 |
|  |  |  |  |  |  |  |  |  |  |
| **Supplementary Table 2 continue** Selected probe/gene (s) with their original source and coefficient regression calculated using the gene expression values of Versteeg dataset | | | | | | | | | |
| Probe | Gene | Medline Search | 144 gene signature (Oberthuer et al., 2006) | 55 gene signature (Asgharzadeh et al., 2006) | 59 gene signature (Vermeulen et al., 2009) | 32 gene signature (Fardin et al, 2010) | 14 gene signature (Asgharzadeh et al., 2012) | 101 genes from NB cell lines treated with retinoic acid (GSE9169) | Coefficient regression |
| 208386_x_at | DMC1 | + |  |  |  |  |  |  | 0.064 |
| 208382_s_at | DMC1 | + |  |  |  |  |  |  | -0.006 |
| 239059_at | DNAH1 |  |  | + |  |  |  |  | -0.004 |
| 226281_at | DNER |  | + |  |  |  |  |  | -0.288 |
| 201431_s_at | DPYSL3 |  |  |  | + |  |  |  | -1.502 |
| 215810_x_at | DST | + |  |  |  |  |  |  | 0.398 |
| 218585_s_at | DTL |  | + |  |  |  |  | + | 0.345 |
| 222680_s_at | DTL |  |  |  |  |  |  | + | 0.355 |
| 228033_at | E2F7 |  |  |  |  |  |  | + | 0.533 |
| 219914_at | ECEL1 | + |  |  | + |  |  |  | -0.056 |
| 223046_at | EGLN1 |  |  |  |  | + |  |  | -0.089 |
| 219232_s_at | EGLN3 |  |  |  |  | + |  |  | -0.100 |
| 201303_at | EIF4A3 | + |  |  |  |  |  |  | -0.130 |
| 206051_at | ELAVL4 |  |  |  | + |  |  |  | -0.523 |
| 210839_s_at | ENPP2 |  |  |  |  |  |  | + | -0.190 |
| 209392_at | ENPP2 |  |  |  |  |  |  | + | -0.155 |
| 1562509_at | ENSG00000251011 | |  |  |  |  |  | + | 0.200 |
| 200878_at | EPAS1 | + |  |  |  |  |  |  | 0.166 |
| 206710_s_at | EPB41L3 |  |  |  | + |  |  |  | -0.046 |
| 227449_at | EPHA4 |  |  |  |  |  |  | + | -0.100 |
| 237939_at | EPHA5 |  |  |  | + |  |  |  | -0.167 |
| 203464_s_at | EPN2 |  |  |  | + |  |  |  | -1.047 |
| 235588_at | ESCO2 |  | + |  |  |  |  |  | 0.267 |
| 209214_s_at | EWSR1 | + |  |  |  |  |  |  | -0.184 |
| 204603_at | EXO1 |  | + |  |  |  |  |  | 0.649 |
| 204363_at | F3 | + |  |  |  |  |  |  | 0.056 |
| 220942_x_at | FAM162A |  |  |  |  | + |  |  | 0.527 |
| 225687_at | FAM83D |  | + |  |  |  |  |  | 0.614 |
| 232067_at | FAXC |  |  | + |  |  |  |  | -0.495 |
| 211623_s_at | FBL |  | + |  |  |  |  |  | 0.469 |
| 202766_s_at | FBN1 |  |  |  |  |  |  | + | 0.021 |
| 204006_s_at | FCGR3A,FCGR3B | |  |  |  |  | + |  | 0.010 |
| 1556033_at | FLJ39739 |  | + |  |  |  |  |  | -0.380 |
| 239005_at | FLJ39739 |  | + |  |  |  |  |  | -0.256 |
| 209189_at | FOS | + |  |  |  |  |  |  | 0.091 |
| 229844_at | FOXP1 |  |  | + |  |  |  |  | -1.363 |
| 230645_at | FRMD3 |  |  |  |  |  |  | + | -0.309 |
|  |  |  |  |  |  |  |  |  |  |
| **Supplementary Table 2 continue**. Selected probe/gene (s) with their original source and coefficient regression calculated using the gene expression values of Versteeg dataset | | | | | | | | | |
| Probe | Gene | Medline Search | 144 gene signature (Oberthuer et al., 2006) | 55 gene signature (Asgharzadeh et al., 2006) | 59 gene signature (Vermeulen et al., 2009) | 32 gene signature (Fardin et al, 2010) | 14 gene signature (Asgharzadeh et al., 2012) | 101 genes from NB cell lines treated with retinoic acid (GSE9169) | Coefficient regression |
| 204948_s_at | FST |  |  |  |  |  |  | + | -0.072 |
| 243037_at | FUBP1 |  |  | + |  |  |  |  | -0.590 |
| 238551_at | FUT11 |  |  |  |  | + |  |  | 0.076 |
| 210105_s_at | FYN |  |  |  | + |  |  |  | -0.779 |
| 208869_s_at | GABARAPL1 |  | + |  |  |  |  |  | -0.061 |
| 204471_at | GAP43 | + |  |  |  |  |  | + | -0.365 |
| 216963_s_at | GAP43 | + |  |  |  |  |  |  | -0.379 |
| 216967_at | GAP43 | + |  |  |  |  |  |  | -0.492 |
| 238076_at | GATAD2B |  |  | + |  |  |  |  | -0.183 |
| 221577_x_at | GDF15 |  |  |  |  |  |  | + | 0.271 |
| 229259_at | GFAP | + |  |  |  |  |  |  | 0.522 |
| 203540_at | GFAP | + |  |  |  |  |  |  | 0.252 |
| 214479_at | GFRA3 |  |  | + |  |  | + |  | -0.031 |
| 214431_at | GMPS |  | + |  |  |  |  |  | 0.567 |
| 209576_at | GNAI1 |  |  | + |  |  |  |  | -0.730 |
| 227692_at | GNAI1 |  | + | + |  |  |  |  | -0.739 |
| 200746_s_at | GNB1 |  |  |  | + |  |  |  | -0.776 |
| 224634_at | GPATCH4 |  |  |  |  |  | + |  | 0.992 |
| 219898_at | GPR85 |  |  | + |  |  |  |  | -0.289 |
| 203632_s_at | GPRC5B |  |  |  |  |  |  | + | 0.614 |
| 218469_at | GREM1 |  |  |  |  |  |  | + | 0.064 |
| 218468_s_at | GREM1 |  |  |  |  |  |  | + | 0.034 |
| 210411_s_at | GRIN2B | + |  |  |  |  |  |  | 0.088 |
| 236697_at | H29626 | + |  |  |  |  |  |  | -0.475 |
| 227085_at | H2AFV |  |  | + |  |  | + |  | -0.532 |
| 211222_s_at | HAP1 |  |  | + |  |  |  |  | -0.330 |
| 215584_at | HECW1 | + |  |  |  |  |  |  | 0.547 |
| 210331_at | HECW1 | + |  |  |  |  |  |  | -0.284 |
| 232080_at | HECW2 |  |  |  |  |  |  | + | -0.761 |
| 225981_at | HID1 | + |  |  |  |  |  |  | -0.297 |
| 244593_at | HID1 | + |  |  |  |  |  |  | -0.061 |
| 200989_at | HIF1A | + |  |  |  |  |  |  | 0.646 |
| 1556069_s_at | HIF3A | + |  |  |  |  |  |  | 0.285 |
| 209398_at | HIST1H1C |  | + |  |  |  |  |  | 0.283 |
| 212642_s_at | HIVEP2 |  | + |  | + |  |  |  | -0.430 |
| 235122_at | HIVEP3 |  |  | + |  |  |  |  | -0.263 |
| 208808_s_at | HMGB2 |  | + |  |  |  |  |  | 0.993 |
|  |  |  |  |  |  |  |  |  |  |
| **Supplementary Table 2 continue**. Selected probe/gene (s) with their original source and coefficient regression calculated using the gene expression values of Versteeg dataset | | | | | | | | | |
| Probe | Gene | Medline Search | 144 gene signature (Oberthuer et al., 2006) | 55 gene signature (Asgharzadeh et al., 2006) | 59 gene signature (Vermeulen et al., 2009) | 32 gene signature (Fardin et al, 2010) | 14 gene signature (Asgharzadeh et al., 2012) | 101 genes from NB cell lines treated with retinoic acid (GSE9169) | Coefficient regression |
| 206858_s_at | HOXC6 |  |  | + |  |  |  |  | -0.126 |
| 231936_at | HOXC9 | + | + |  |  |  |  |  | -0.186 |
| 231906_at | HOXD8 |  |  |  |  |  |  | + | 0.303 |
| 206864_s_at | HRK |  |  | + |  |  |  |  | -0.211 |
| 235892_at | Hs.125166 |  |  |  |  |  |  | + | 0.355 |
| 243023_at | Hs.27996 |  |  | + |  |  |  |  | -0.590 |
| 232512_at | Hs.435274 | + |  | + |  |  |  |  | 0.236 |
| 232396_at | Hs.655961 |  |  | + |  |  |  |  | 0.492 |
| 237295_at | Hs.656974 | + |  |  |  |  |  |  | 0.156 |
| 243743_at | Hs.669003 | + |  |  |  |  |  |  | -0.031 |
| 215727_x_at | Hs.669954 |  |  | + |  |  |  |  | 0.332 |
| 243567_at | Hs.670807 | + |  |  |  |  |  |  | 0.575 |
| 237305_at | Hs.674599 | + |  |  |  |  |  |  | 0.473 |
| 216062_at | Hs.675304 | + |  |  |  |  |  |  | 0.113 |
| 240751_at | Hs.680066 |  |  | + |  |  |  |  | 0.018 |
| 227061_at | Hs.86538 |  |  |  |  |  |  | + | 0.035 |
| 229714_at | HS6ST3 |  | + |  |  |  |  |  | -0.249 |
| 206638_at | HTR2B |  |  |  |  |  |  | + | -0.021 |
| 226757_at | IFIT2 |  |  |  |  |  |  | + | -0.101 |
| 225330_at | IGF1R |  |  |  |  | + |  |  | -0.544 |
| 210095_s_at | IGFBP3 |  |  |  |  | + |  |  | 0.226 |
| 228375_at | IGSF11 |  |  |  |  |  |  | + | 0.007 |
| 207433_at | IL10 |  |  |  |  |  | + |  | 0.135 |
| 226333_at | IL6R |  |  |  |  |  | + |  | 0.158 |
| 211506_s_at | IL8 |  |  |  |  |  |  | + | 0.019 |
| 230719_at | ING3 |  |  | + |  |  |  |  | -0.406 |
| 202794_at | INPP1 |  |  |  | + |  |  |  | -0.607 |
| 240841_at | INSM2 |  |  |  |  |  |  | + | -0.138 |
| 222240_s_at | ISYNA1 |  |  |  |  |  |  | + | 0.537 |
| 214660_at | ITGA1 |  |  |  |  |  |  | + | -0.088 |
| 226731_at | ITGA1 |  |  |  |  |  |  | + | -0.054 |
| 213416_at | ITGA4 |  |  |  |  |  |  | + | 0.145 |
| 212689_s_at | KDM3A |  |  |  |  | + |  |  | 0.531 |
| 230249_at | KHDRBS3 |  |  | + |  |  |  |  | -0.345 |
| 236641_at | KIF14 |  |  |  |  |  |  | + | 0.504 |
| 209234_at | KIF1B |  | + |  |  |  |  |  | -0.643 |
| 205051_s_at | KIT | + |  |  |  |  |  |  | 0.186 |
|  |  |  |  |  |  |  |  |  |  |
| **Supplementary Table 2 continue**. Selected probe/gene (s) with their original source and coefficient regression calculated using the gene expression values of Versteeg dataset | | | | | | | | | |
| Probe | Gene | Medline Search | 144 gene signature (Oberthuer et al., 2006) | 55 gene signature (Asgharzadeh et al., 2006) | 59 gene signature (Vermeulen et al., 2009) | 32 gene signature (Fardin et al, 2010) | 14 gene signature (Asgharzadeh et al., 2012) | 101 genes from NB cell lines treated with retinoic acid (GSE9169) | Coefficient regression |
| 1555832_s_at | KLF6 | + |  |  |  |  |  |  | -0.469 |
| 205266_at | LIF | + |  |  |  |  |  |  | 0.258 |
| 229280_s_at | LINC00340 |  | + |  |  |  |  |  | -0.202 |
| 239799_at | LINC00476 |  |  | + |  |  |  |  | -0.500 |
| 204424_s_at | LMO3 | + |  |  |  |  |  |  | 0.232 |
| 231348_s_at | LMO3 | + |  |  |  |  |  |  | 0.124 |
| 217378_x_at | LOC100130100 | |  | + |  |  |  |  | 0.107 |
| 225342_at | LOC100507855,AK4 | |  |  |  | + |  |  | -0.219 |
| 215016_x_at | LOC100652766,DST | + | + |  |  |  |  |  | -1.392 |
| 232794_at | LOC153682 |  |  | + |  |  |  |  | -0.602 |
| 214162_at | LOC284244 |  |  | + |  |  |  |  | -0.452 |
| 1560692_at | LOC285878 |  | + |  |  |  |  |  | -0.280 |
| 228564_at | LOC375295 |  |  |  |  |  |  | + | 0.491 |
| 213388_at | LOC728802,PDE4DIP | | + |  | + |  |  |  | -0.974 |
| 212390_at | LOC728802,PDE4DIP | | + |  |  |  |  |  | -0.484 |
| 200785_s_at | LRP1 | + |  |  |  |  |  |  | 0.101 |
| 209841_s_at | LRRN3 | + |  |  |  |  |  |  | -0.082 |
| 209840_s_at | LRRN3 | + |  |  |  |  |  |  | -0.059 |
| 203362_s_at | MAD2L1 |  | + |  |  |  |  |  | 0.340 |
| 1554768_a_at | MAD2L1 |  | + |  |  |  |  |  | 0.067 |
| 224650_at | MAL2 |  |  |  |  |  |  | + | -0.016 |
| 203266_s_at | MAP2K4 |  |  |  | + |  |  |  | -0.930 |
| 215471_s_at | MAP7 | + | + |  |  |  |  |  | -0.640 |
| 202890_at | MAP7 | + | + |  | + |  |  |  | -0.303 |
| 202889_x_at | MAP7 |  | + |  |  |  |  |  | -0.326 |
| 225379_at | MAPT |  | + |  | + | + |  |  | -0.465 |
| 209072_at | MBP | + |  |  |  |  |  |  | -0.031 |
| 226797_at | MBTD1 | + |  |  |  |  |  |  | -0.384 |
| 201620_at | MBTPS1 | + |  |  |  |  |  |  | -0.957 |
| 220651_s_at | MCM10 |  |  |  |  |  |  | + | 0.427 |
| 202107_s_at | MCM2 |  |  |  | + |  |  |  | 0.315 |
| 201930_at | MCM6 |  | + |  |  |  |  |  | 0.562 |
| 238977_at | MCM6 |  | + |  |  |  |  |  | 0.311 |
| 209035_at | MDK |  |  |  |  |  |  | + | 0.299 |
| 242172_at | MEIS1 | + |  |  |  |  |  |  | -0.485 |
| 204069_at | MEIS1 | + |  |  |  |  |  |  | -0.385 |
| 218883_s_at | MLF1IP |  | + |  |  |  |  |  | 0.293 |
|  |  |  |  |  |  |  |  |  |  |
| **Supplementary Table 2 continue**. Selected probe/gene (s) with their original source and coefficient regression calculated using the gene expression values of Versteeg dataset | | | | | | | | | |
| Probe | Gene | Medline Search | 144 gene signature (Oberthuer et al., 2006) | 55 gene signature (Asgharzadeh et al., 2006) | 59 gene signature (Vermeulen et al., 2009) | 32 gene signature (Fardin et al, 2010) | 14 gene signature (Asgharzadeh et al., 2012) | 101 genes from NB cell lines treated with retinoic acid (GSE9169) | Coefficient regression |
| 204475_at | MMP1 |  |  |  |  |  |  | + | 0.173 |
| 203878_s_at | MMP11 |  |  |  |  |  |  | + | 0.186 |
| 223700_at | MND1 |  | + |  |  |  |  |  | 0.421 |
| 205413_at | MPPED2 |  |  |  |  |  |  | + | -0.124 |
| 208787_at | MRPL3 |  | + |  | + |  |  |  | 1.046 |
| 223172_s_at | MTFP1 |  |  |  |  | + |  |  | 0.617 |
| 203037_s_at | MTSS1 |  |  |  | + |  |  |  | -0.772 |
| 202364_at | MXI1 |  |  |  |  | + |  |  | -0.313 |
| 211377_x_at | MYCN | + |  |  |  |  |  |  | 0.058 |
| 242026_at | MYCN | + |  |  |  |  |  |  | 0.096 |
| 234376_at | MYCN | + |  |  |  |  |  |  | 0.077 |
| 209756_s_at | MYCN | + |  |  |  |  |  |  | 0.040 |
| 209757_s_at | MYCN | + |  |  | + |  |  |  | 0.052 |
| 228880_at | NAT8L | + |  |  |  |  |  |  | 0.933 |
| 238032_at | NAV2 |  |  |  |  |  |  | + | 0.212 |
| 212843_at | NCAM1 | + |  |  |  |  |  |  | -0.827 |
| 229799_s_at | NCAM1 | + |  |  |  |  |  |  | 0.045 |
| 205669_at | NCAM2 |  |  |  |  |  |  | + | 0.081 |
| 232390_at | NCAM2 |  |  |  |  |  |  | + | -0.042 |
| 212949_at | NCAPH |  | + |  |  |  |  |  | 0.209 |
| 200632_s_at | NDRG1 |  |  |  |  | + |  |  | 0.135 |
| 33767_at | NEFH | + |  |  |  |  |  |  | 0.074 |
| 221916_at | NEFL | + |  |  |  |  |  |  | 0.159 |
| 204641_at | NEK2 |  | + |  |  |  |  |  | 0.281 |
| 206282_at | NEUROD1 | + |  |  |  |  |  |  | 0.151 |
| 210162_s_at | NFATC1 | + |  |  |  |  |  |  | 0.062 |
| 201146_at | NFE2L2 | + |  |  |  |  |  |  | 0.282 |
| 205858_at | NGFR | + |  |  |  |  |  |  | -0.030 |
| 215228_at | NHLH2 | + |  |  | + |  |  |  | 0.045 |
| 214497_s_at | NHLH2 | + |  |  |  |  |  |  | -0.010 |
| 205004_at | NKRF | + |  |  |  |  |  |  | -0.201 |
| 201577_at | NME1 | + |  |  | + |  |  |  | 0.355 |
| 201268_at | NME2,NME1-NME2 | + |  |  |  |  |  |  | 0.485 |
| 231798_at | NOG |  |  |  |  |  |  | + | 0.234 |
| 206001_at | NPY | + |  |  |  |  |  | + | 0.008 |
| 204105_s_at | NRCAM |  | + |  | + |  |  |  | -0.466 |
| 208605_s_at | NTRK1 | + | + | + | + |  |  |  | -0.170 |
| 236095_at | NTRK2 |  |  |  |  |  |  | + | 0.185 |
| **Supplementary Table 2 continue**. Selected probe/gene (s) with their original source and coefficient regression calculated using the gene expression values of Versteeg dataset | | | | | | | | | |
| Probe | Gene | Medline Search | 144 gene signature (Oberthuer et al., 2006) | 55 gene signature (Asgharzadeh et al., 2006) | 59 gene signature (Vermeulen et al., 2009) | 32 gene signature (Fardin et al, 2010) | 14 gene signature (Asgharzadeh et al., 2012) | 101 genes from NB cell lines treated with retinoic acid (GSE9169) | Coefficient regression |
| 229463_at | NTRK2 |  |  |  |  |  |  | + | 0.115 |
| 221795_at | NTRK2 | + |  |  |  |  |  |  | 0.118 |
| 214680_at | NTRK2 | + |  | + |  |  | + |  | 0.068 |
| 221796_at | NTRK2 |  |  |  |  |  |  | + | -0.007 |
| 215115_x_at | NTRK3 | + |  |  |  |  |  |  | -0.319 |
| 228849_at | NTRK3 | + |  |  |  |  |  |  | 0.353 |
| 217377_x_at | NTRK3 | + |  |  |  |  |  |  | -0.161 |
| 217033_x_at | NTRK3 | + |  |  |  |  |  |  | -0.156 |
| 215025_at | NTRK3 | + |  |  |  |  |  |  | 0.040 |
| 206462_s_at | NTRK3 | + |  |  |  |  |  |  | -0.005 |
| 223381_at | NUF2 |  | + |  |  |  |  |  | 0.525 |
| 232377_at | NXPH1 |  | + |  |  |  |  |  | -0.240 |
| 200790_at | ODC1 |  |  |  | + |  |  |  | 0.587 |
| 202733_at | P4HA2 |  |  |  |  | + |  |  | -0.129 |
| 201013_s_at | PAICS |  | + |  | + |  |  |  | 0.670 |
| 219148_at | PBK |  | + |  |  |  |  |  | 0.315 |
| 212151_at | PBX1 |  |  |  |  |  |  | + | -0.209 |
| 212148_at | PBX1 |  |  |  |  |  |  | + | -0.062 |
| 208620_at | PCBP1 | + |  |  |  |  |  |  | 0.768 |
| 209361_s_at | PCBP4 | + |  |  |  |  |  |  | -0.181 |
| 201202_at | PCNA |  | + |  |  |  |  |  | 0.564 |
| 226452_at | PDK1 |  |  |  |  | + |  |  | 0.493 |
| 210825_s_at | PEBP1 | + |  |  |  |  |  |  | -0.073 |
| 1560359_at | PELO |  |  |  |  |  |  | + | -0.179 |
| 228499_at | PFKFB4 |  |  |  |  | + |  |  | -0.177 |
| 200738_s_at | PGK1 |  |  |  |  | + |  |  | 1.318 |
| 201968_s_at | PGM1 |  |  |  |  | + |  |  | -0.017 |
| 229256_at | PGM2L1 |  | + |  |  |  |  |  | -0.672 |
| 229553_at | PGM2L1 |  | + |  |  |  |  |  | -0.779 |
| 238417_at | PGM2L1 |  |  | + |  |  | + |  | -0.643 |
| 207009_at | PHOX2B | + |  |  |  |  |  |  | -0.321 |
| 212240_s_at | PIK3R1 |  |  |  | + |  |  |  | -0.277 |
| 222150_s_at | PION |  |  |  |  |  |  | + | -0.198 |
| 223551_at | PKIB |  | + |  |  |  |  |  | -0.181 |
| 209318_x_at | PLAGL1 |  |  |  | + |  |  |  | -0.063 |
| 201860_s_at | PLAT |  |  |  | + |  |  | + | 0.057 |
| 200827_at | PLOD1 |  |  |  |  | + |  |  | 0.612 |
| 213241_at | PLXNC1 |  | + |  |  |  |  |  | -0.311 |
| **Supplementary Table 2 continue**. Selected probe/gene (s) with their original source and coefficient regression calculated using the gene expression values of Versteeg dataset | | | | | | | | | |
| Probe | Gene | Medline Search | 144 gene signature (Oberthuer et al., 2006) | 55 gene signature (Asgharzadeh et al., 2006) | 59 gene signature (Vermeulen et al., 2009) | 32 gene signature (Fardin et al, 2010) | 14 gene signature (Asgharzadeh et al., 2012) | 101 genes from NB cell lines treated with retinoic acid (GSE9169) | Coefficient regression |
| 210139_s_at | PMP22 |  | + |  | + |  |  |  | -0.558 |
| 208510_s_at | PPARG |  |  |  |  |  |  | + | 0.138 |
| 204566_at | PPM1D | + |  |  |  |  |  |  | -0.416 |
| 204086_at | PRAME | + |  |  | + |  |  |  | -0.016 |
| 203057_s_at | PRDM2 |  |  |  | + |  |  |  | 0.079 |
| 201923_at | PRDX4 |  | + |  |  |  |  |  | 0.716 |
| 211743_s_at | PRG2 |  |  | + |  |  |  |  | 0.265 |
| 226069_at | PRICKLE1 |  |  |  |  |  |  | + | -0.022 |
| 202741_at | PRKACB |  | + |  | + |  |  |  | -1.099 |
| 202178_at | PRKCZ |  |  |  | + |  |  |  | -0.465 |
| 204304_s_at | PROM1 | + |  |  |  |  |  |  | 0.458 |
| 227192_at | PRRT2 | + |  |  |  |  |  |  | -0.547 |
| 205515_at | PRSS12 |  |  |  |  |  |  | + | 0.043 |
| 213802_at | PRSS12 |  |  |  |  |  |  | + | 0.026 |
| 212805_at | PRUNE2 | + |  |  |  |  |  |  | -0.256 |
| 212806_at | PRUNE2 | + |  |  |  |  |  |  | -0.251 |
| 223536_at | PSD2 |  | + |  |  |  |  |  | -0.358 |
| 208408_at | PTN | + |  |  |  |  |  |  | 0.176 |
| 209466_x_at | PTN | + |  |  |  |  |  |  | -0.144 |
| 211737_x_at | PTN | + | + |  | + |  |  |  | -0.114 |
| 209465_x_at | PTN | + |  |  |  |  |  |  | -0.083 |
| 236456_at | PTPN5 |  |  |  |  |  | + |  | 0.044 |
| 212588_at | PTPRC | + |  |  |  |  |  |  | 0.085 |
| 200636_s_at | PTPRF |  |  |  | + |  |  |  | 0.019 |
| 208300_at | PTPRH |  | + |  | + |  |  |  | -0.317 |
| 203029_s_at | PTPRN2 |  |  |  | + |  |  |  | -0.169 |
| 203554_x_at | PTTG1 |  | + |  |  |  |  |  | 0.599 |
| 205174_s_at | QPCT |  |  |  | + |  |  |  | -0.057 |
| 209089_at | RAB5A | + |  |  |  |  |  |  | -0.890 |
| 211961_s_at | RAB7A | + |  |  |  |  |  |  | -0.105 |
| 202483_s_at | RANBP1 |  | + |  |  |  |  |  | 0.451 |
| 205080_at | RARB |  |  |  |  |  |  | + | 0.197 |
| 208530_s_at | RARB |  |  |  |  |  |  | + | 0.037 |
| 206392_s_at | RARRES1 |  |  |  |  |  |  | + | 0.115 |
| 201394_s_at | RBM5 | + |  |  |  |  |  |  | -0.846 |
| 201395_at | RBM5 | + |  |  |  |  |  |  | -0.663 |
| 204023_at | RFC4 |  | + |  |  |  |  |  | 0.254 |
| 210258_at | RGS13 |  |  |  |  |  |  | + | -0.016 |
| **Supplementary Table 2 continue**. Selected probe/gene (s) with their original source and coefficient regression calculated using the gene expression values of Versteeg dataset | | | | | | | | | |
| Probe | Gene | Medline Search | 144 gene signature (Oberthuer et al., 2006) | 55 gene signature (Asgharzadeh et al., 2006) | 59 gene signature (Vermeulen et al., 2009) | 32 gene signature (Fardin et al, 2010) | 14 gene signature (Asgharzadeh et al., 2012) | 101 genes from NB cell lines treated with retinoic acid (GSE9169) | Coefficient regression |
| 206518_s_at | RGS9 |  | + |  |  |  |  |  | -0.220 |
| 235948_at | RIMKLA | + |  |  |  |  |  |  | 0.128 |
| 208924_at | RNF11 |  | + |  |  |  |  |  | -0.568 |
| 213194_at | ROBO1 | + |  |  |  |  |  |  | -0.061 |
| 201406_at | RPL36A-HNRNPH2,RPL36A | | + |  |  |  |  |  | 0.725 |
| 201477_s_at | RRM1 |  | + |  |  |  |  |  | 0.599 |
| 201890_at | RRM2 |  | + |  |  |  |  |  | 0.213 |
| 209686_at | S100B | + |  |  |  |  |  |  | -0.203 |
| 204035_at | SCG2 |  | + |  | + |  |  |  | -0.465 |
| 208578_at | SCN10A | + |  |  |  |  |  |  | 0.226 |
| 220791_x_at | SCN11A | + |  |  |  |  |  |  | 0.275 |
| 210853_at | SCN11A | + |  |  |  |  |  |  | 0.185 |
| 224029_x_at | SCN11A | + |  |  |  |  |  |  | -0.019 |
| 210383_at | SCN1A | + |  |  |  |  |  |  | 0.071 |
| 206381_at | SCN2A | + |  |  |  |  |  |  | -0.243 |
| 210432_s_at | SCN3A | + |  |  |  |  |  |  | -0.457 |
| 204722_at | SCN3B |  |  | + |  |  |  |  | -0.888 |
| 206981_at | SCN4A | + |  |  |  |  |  |  | 0.010 |
| 207413_s_at | SCN5A | + |  |  |  |  |  |  | 0.321 |
| 228504_at | SCN7A |  | + |  |  |  |  |  | -0.169 |
| 207864_at | SCN7A | + |  |  |  |  |  |  | -0.151 |
| 207049_at | SCN8A | + |  |  |  |  |  |  | 0.044 |
| 229199_at | SCN9A | + |  |  |  |  |  |  | -0.375 |
| 206950_at | SCN9A | + |  |  |  |  |  |  | -0.313 |
| 213151_s_at | SEPT7 | + |  |  |  |  |  |  | -1.159 |
| 244508_at | SEPT7 | + |  |  |  |  |  |  | 0.071 |
| 224762_at | SERINC2 |  | + |  |  |  |  |  | -0.121 |
| 211429_s_at | SERPINA1 |  |  |  |  |  |  | + | -0.157 |
| 202833_s_at | SERPINA1 |  |  |  |  |  |  | + | -0.065 |
| 202376_at | SERPINA3 |  |  |  |  |  |  | + | 0.017 |
| 212268_at | SERPINB1 |  |  |  |  |  |  | + | -0.056 |
| 222986_s_at | SHISA5 | + |  |  |  |  |  |  | 0.142 |
| 200657_at | SLC25A5 |  | + |  | + |  |  |  | 0.956 |
| 202499_s_at | SLC2A3 |  |  |  |  | + |  |  | 0.174 |
| 224596_at | SLC44A1 | + |  |  |  |  |  |  | 0.294 |
| 202219_at | SLC6A8 |  |  |  | + |  |  |  | 0.391 |
| 227367_at | SLCO3A1 |  | + |  |  |  |  |  | -0.368 |
| 211989_at | SMARCE1 |  |  | + |  |  |  |  | -0.468 |
| **Supplementary Table 2 continue**. Selected probe/gene (s) with their original source and coefficient regression calculated using the gene expression values of Versteeg dataset | | | | | | | | | |
| Probe | Gene | Medline Search | 144 gene signature (Oberthuer et al., 2006) | 55 gene signature (Asgharzadeh et al., 2006) | 59 gene signature (Vermeulen et al., 2009) | 32 gene signature (Fardin et al, 2010) | 14 gene signature (Asgharzadeh et al., 2012) | 101 genes from NB cell lines treated with retinoic acid (GSE9169) | Coefficient regression |
| 223276_at | SMIM3 |  |  |  |  |  |  | + | 0.195 |
| 204953_at | SNAP91 | + |  |  |  |  |  |  | -0.785 |
| 205443_at | SNAPC1 |  |  |  | + |  |  |  | 0.352 |
| 219511_s_at | SNCAIP |  |  |  |  |  |  | + | 0.122 |
| 203316_s_at | SNRPE |  | + |  |  |  |  |  | 1.297 |
| 209842_at | SOX10 | + |  |  |  |  |  |  | -0.272 |
| 201417_at | SOX4 |  |  | + |  |  |  |  | -0.494 |
| 213665_at | SOX4 |  |  | + |  |  |  |  | 0.073 |
| 209891_at | SPC25 |  | + |  |  |  |  |  | 0.291 |
| 224144_at | SPTBN4 |  |  | + |  |  |  |  | -0.179 |
| 202817_s_at | SS18 | + |  |  |  |  |  |  | -0.153 |
| 200957_s_at | SSRP1 |  | + |  |  |  |  |  | 1.224 |
| 208482_at | SSTR1 | + |  |  |  |  |  |  | 0.301 |
| 235591_at | SSTR1 | + |  |  |  |  |  |  | 0.120 |
| 217455_s_at | SSTR2 | + |  |  |  |  |  |  | -0.213 |
| 239031_at | SSTR2 | + |  |  |  |  |  |  | 0.153 |
| 214491_at | SSTR3 | + |  |  |  |  |  |  | -0.386 |
| 214556_at | SSTR4 | + |  |  |  |  |  |  | 0.237 |
| 214555_at | SSTR5 | + |  |  |  |  |  |  | 0.249 |
| 207524_at | ST7,ST7-OT3 | |  | + |  |  |  |  | 0.187 |
| 202693_s_at | STK17A |  | + |  |  |  |  |  | 0.132 |
| 212111_at | STX12 |  | + |  |  |  |  |  | -0.784 |
| 206322_at | SYN3 | + | + |  |  |  |  |  | -0.178 |
| 213200_at | SYP | + |  |  |  |  |  |  | -0.113 |
| 209938_at | TADA2A |  |  | + |  |  |  |  | 0.009 |
| 211052_s_at | TBCD | + |  |  |  |  |  |  | 0.240 |
| 201174_s_at | TERF2IP | + |  |  |  |  |  |  | -1.171 |
| 207199_at | TERT | + |  |  |  |  |  |  | 0.010 |
| 202720_at | TES |  |  |  |  |  |  | + | 0.186 |
| 214063_s_at | TF | + |  |  |  |  |  |  | -0.361 |
| 203400_s_at | TF | + |  |  |  |  |  |  | -0.091 |
| 220109_at | TF | + |  |  |  |  |  |  | -0.026 |
| 214064_at | TF | + |  |  |  |  |  |  | 0.019 |
| 215686_x_at | TFAP2B | + |  |  |  |  |  |  | -0.048 |
| 214451_at | TFAP2B | + |  |  |  |  |  |  | 0.010 |
| 208691_at | TFRC | + |  |  |  |  |  |  | 0.170 |
| 208291_s_at | TH | + |  |  |  |  |  |  | -0.031 |
| 230380_at | THAP2 |  |  | + |  |  | + |  | 0.010 |
| **Supplementary Table 2 continue**. Selected probe/gene (s) with their original source and coefficient regression calculated using the gene expression values of Versteeg dataset | | | | | | | | | |
| Probe | Gene | Medline Search | 144 gene signature (Oberthuer et al., 2006) | 55 gene signature (Asgharzadeh et al., 2006) | 59 gene signature (Vermeulen et al., 2009) | 32 gene signature (Fardin et al, 2010) | 14 gene signature (Asgharzadeh et al., 2012) | 101 genes from NB cell lines treated with retinoic acid (GSE9169) | Coefficient regression |
| 203887_s_at | THBD |  |  |  |  |  |  | + | 0.250 |
| 1554408_a_at | TK1 |  | + |  |  |  |  |  | 0.285 |
| 223557_s_at | TMEFF2 |  |  |  |  |  |  | + | -0.299 |
| 226529_at | TMEM106B |  | + |  |  |  |  |  | -1.583 |
| 218834_s_at | TMEM132A | + |  |  |  |  |  |  | 0.263 |
| 219895_at | TMEM255A |  | + |  |  |  |  |  | -0.123 |
| 219701_at | TMOD2 |  |  | + |  |  |  |  | -0.086 |
| 207113_s_at | TNF | + |  |  |  |  |  |  | -0.004 |
| 219423_x_at | TNFRSF25 |  |  |  | + |  |  |  | -0.159 |
| 207196_s_at | TNIP1 |  |  |  |  | + |  |  | 0.936 |
| 226198_at | TOM1L2 |  | + |  |  |  |  |  | -0.329 |
| 232546_at | TP73 | + |  |  |  |  |  |  | 0.412 |
| 220804_s_at | TP73 | + |  |  |  |  |  |  | -0.003 |
| 213011_s_at | TPI1 |  |  |  |  | + |  |  | 0.781 |
| 202154_x_at | TUBB3 | + |  |  |  |  |  |  | -0.649 |
| 213476_x_at | TUBB3 | + |  |  |  |  |  |  | -0.648 |
| 201010_s_at | TXNIP |  |  |  |  | + |  |  | 0.193 |
| 202589_at | TYMS |  | + |  | + |  |  |  | 0.549 |
| 202954_at | UBE2C |  | + |  |  |  |  |  | 0.347 |
| 201387_s_at | UCHL1 | + |  |  |  |  |  |  | -0.699 |
| 204062_s_at | ULK2 |  |  |  | + |  |  |  | -1.254 |
| 231325_at | UNC5D |  |  |  |  |  |  | + | -0.172 |
| 210512_s_at | VEGFA | + |  |  |  |  |  |  | 0.093 |
| 211527_x_at | VEGFA |  |  |  |  | + |  |  | -0.046 |
| 201426_s_at | VIM | + |  |  |  |  |  |  | 0.391 |
| 211598_x_at | VIPR2 | + |  |  |  |  |  |  | 0.089 |
| 203856_at | VRK1 |  | + |  |  |  |  |  | 0.086 |
| 218851_s_at | WDR33,SFT2D3 | |  | + |  |  |  |  | 0.014 |
| 201296_s_at | WSB1 |  | + |  | + |  |  |  | -0.885 |
| 210561_s_at | WSB1 |  | + |  |  |  |  |  | -0.628 |
| 237802_at | XKR4 |  | + |  |  |  |  |  | -0.067 |
| 225629_s_at | ZBTB4 |  | + |  |  |  |  |  | -0.893 |
| 229551_x_at | ZNF367 |  |  |  |  |  |  | + | 0.364 |
| 218149_s_at | ZNF395 |  |  |  |  | + |  |  | 0.865 |
| 214761_at | ZNF423 | + |  |  |  |  |  |  | -0.161 |
| 206059_at | ZNF91 |  |  | + |  |  |  |  | -0.517 |

| **Supplementary Table 3.** Results of the retinoic acid induced neuroblastoma cell lines from microarray data analysis | | | | | | | | |
| --- | --- | --- | --- | --- | --- | --- | --- | --- |
|  |  |  | **Fold change** | |  |  |  |  |
| **N** | **Genes** | **Probe ID** | **6 hours** | **1day** | **2 days** | **3 days** | **5 days** |  |
| *SH-SY5Y ATCC* | |  |  |  |  |  |  | **Delta-value >1.821** |
| 1 | **CYP26A1** | 206424_at | 5.26 | 8.56 | 8.85 | 8.81 | 8.63 | 4.85 |
| 2 | **CYP26B1** | 219825_at | 3.72 | 8.84 | 9.27 | 9.25 | 9.26 | 4.42 |
| 3 | **CALCA** | 217561_at | 0.95 | 4.68 | 6.86 | 7.58 | 7.99 | 3.21 |
| 4 | **DLK1** | 209560_s_at | -0.26 | -2.04 | -3.90 | -4.75 | -5.52 | 2.79 |
| 5 | **DHRS3** | 202481_at | 3.40 | 4.64 | 4.75 | 4.85 | 4.81 | 2.40 |
| 6 | **ENPP2** | 210839_s_at | 1.15 | 3.20 | 4.23 | 4.29 | 4.53 | 2.28 |
| 7 | **NTRK2** | 236095_at | 1.43 | 3.22 | 4.16 | 4.66 | 5.09 | 2.16 |
| 8 | **NTRK2** | 229463_at | 1.91 | 3.59 | 4.45 | 4.82 | 5.13 | 2.16 |
| 9 | **RGS13** | 210258_at | 0.32 | 3.59 | 5.08 | 5.59 | 6.31 | 2.14 |
| 10 | **CALCA** | 217495_x_at | 0.11 | 1.64 | 2.92 | 3.51 | 3.75 | 2.11 |
| 11 | **SNCAIP** | 219511_s_at | 1.05 | 3.56 | 4.11 | 3.84 | 3.41 | 2.04 |
| 12 | **CCBE1** | 229641_at | 2.43 | 4.49 | 4.48 | 3.99 | 2.63 | 2.04 |
| 13 | **INSM2** | 240841_at | -2.20 | -3.94 | -4.43 | -4.56 | -4.70 | 2.02 |
| 14 | **CRABP2** | 202575_at | 2.95 | 4.56 | 4.62 | 4.50 | 4.27 | 2.01 |
| 15 | **ENPP2** | 209392_at | 1.22 | 3.28 | 4.17 | 4.28 | 4.47 | 2.00 |
| 16 | **CALCB** | 214636_at | 0.75 | 3.21 | 4.06 | 4.39 | 4.34 | 1.99 |
| 17 | **HTR2B** | 206638_at | 1.32 | 3.55 | 3.41 | 3.59 | 4.32 | 1.98 |
| 18 | **UNC5D** | 231325_at | -0.44 | -2.43 | -3.54 | -4.39 | -4.54 | 1.94 |
| 19 | **C4orf6** | 207241_at | 1.53 | 4.02 | 4.51 | 4.48 | 4.19 | 1.90 |
| 20 | N/A | 235892_at | 1.05 | 3.78 | 4.85 | 4.95 | 5.23 | 1.89 |
| 21 | **NTRK2** | 221796_at | 2.07 | 4.08 | 4.36 | 4.09 | 3.83 | 1.85 |
| 22 | **NCAM2** | 205669_at | -0.31 | 1.71 | 2.81 | 3.39 | 3.93 | 1.82 |
| 23 | **CLMN** | 213839_at | 2.48 | 3.16 | 3.97 | 4.27 | 4.21 | 1.82 |
| *SH-SY5Y ECACC* | |  |  |  |  |  |  | **Delta-value >2.453** |
| 1 | **CYP26B1** | 219825_at | 3.55 | 8.47 | 9.60 | 9.63 | 9.85 | 7.17 |
| 2 | **CYP26A1** | 206424_at | 6.55 | 8.67 | 9.10 | 9.12 | 9.06 | 6.25 |
| 3 | **CFI** | 1555564_a_at | 0.83 | 4.31 | 5.82 | 6.70 | 6.62 | 4.00 |
| 4 | **MPPED2** | 205413_at | 2.02 | 4.05 | 5.22 | 5.92 | 6.70 | 3.78 |
| 5 | **CFI** | 203854_at | 0.91 | 4.36 | 5.80 | 6.59 | 6.57 | 3.51 |
| 6 | **C4orf6** | 207241_at | 4.04 | 5.35 | 5.30 | 5.12 | 4.30 | 3.33 |
| 7 | **NPY** | 206001_at | -0.08 | -0.81 | -1.49 | -2.05 | -4.96 | 3.24 |
| 8 | **HOXD8** | 231906_at | 1.40 | 1.91 | 2.30 | 2.75 | 2.94 | 2.89 |
| 9 | **DTL** | 218585_s_at | -0.19 | -0.29 | -0.43 | -1.03 | -2.83 | 2.67 |
| 10 | N/A | 1562509_at | -1.06 | -2.52 | -2.99 | -2.95 | -3.23 | 2.63 |
| 11 | **C11orf93** | 228338_at | 2.03 | 2.79 | 3.47 | 3.86 | 4.26 | 2.63 |
| 12 | **IGSF11** | 228375_at | 1.26 | 2.24 | 3.11 | 3.70 | 4.02 | 2.61 |
| 13 | **C5orf62** | 223276_at | 1.29 | 2.26 | 2.42 | 2.39 | 1.42 | 2.60 |
| 14 | **LOC375295** | 228564_at | 1.49 | 2.19 | 2.45 | 2.62 | 2.82 | 2.53 |
| 15 | **MMP11** | 203878_s_at | 0.10 | 0.48 | 1.02 | 1.45 | 3.10 | 2.53 |
| 16 | **PPARG** | 208510_s_at | 1.24 | 3.22 | 3.98 | 4.32 | 4.52 | 2.51 |
| 17 | **GAP43** | 204471_at | -0.42 | -0.93 | -1.63 | -1.96 | -4.10 | 2.45 |
| **Supplementary Table 3 continue.** Results of the retinoic acid induced neuroblastoma cell lines from microarray data analysis | | | | | | | | |
|  |  |  | **Fold change** | |  |  |  |  |
| **N** | **Genes** | **Probe ID** | **6 hours** | **1day** | **2 days** | **3 days** | **5 days** |  |
| *SK-N-SH* | |  |  |  |  |  |  | **Delta-value > 3.708** |
| 1 | **CYP26B1** | 219825_at | 5.03 | 2.70 | 8.17 | 8.47 | 8.84 | 10.61 |
| 2 | **CRABP2** | 202575_at | 3.88 | 0.91 | 4.87 | 5.07 | 4.95 | 7.83 |
| 3 | **COL15A1** | 203477_at | -0.21 | -1.06 | -2.97 | -4.14 | -5.31 | 7.45 |
| 4 | **NOG** | 231798_at | -1.65 | -1.19 | -3.48 | -4.31 | -4.62 | 6.92 |
| 5 | **SERPINA3** | 202376_at | 0.42 | 1.50 | 3.64 | 5.11 | 5.81 | 6.83 |
| 6 | **GREM1** | 218469_at | -1.50 | -1.23 | -3.59 | -4.92 | -6.53 | 6.73 |
| 7 | **COL1A2** | 202404_s_at | -0.50 | -2.32 | -3.68 | -3.59 | -3.54 | 6.56 |
| 8 | **TMEFF2** | 223557_s_at | 0.83 | 2.49 | 4.09 | 4.04 | 3.94 | 6.17 |
| 9 | **GREM1** | 218468_s_at | -1.35 | -1.51 | -3.62 | -4.91 | -6.62 | 6.12 |
| 10 | **CYP24A1** | 206504_at | 2.75 | 1.38 | 5.09 | 5.10 | 5.54 | 5.97 |
| 11 | **ISYNA1** | 222240_s_at | 0.27 | 0.94 | 1.51 | 2.07 | 2.63 | 5.76 |
| 12 | N/A | 238032_at | 2.72 | 0.58 | 3.37 | 3.57 | 3.52 | 5.67 |
| 13 | **MAL2** | 224650_at | -0.02 | 1.42 | 2.89 | 3.55 | 4.40 | 5.67 |
| 14 | **CD177** | 219669_at | -0.07 | -0.09 | 0.56 | 2.41 | 4.77 | 5.34 |
| 15 | **RARB** | 205080_at | 2.57 | 0.22 | 2.92 | 3.12 | 3.16 | 5.33 |
| 16 | **MMP1** | 204475_at | -0.31 | -1.75 | -4.40 | -5.63 | -6.16 | 5.33 |
| 17 | **CD55** | 201926_s_at | 0.58 | 1.57 | 3.03 | 3.17 | 3.81 | 5.08 |
| 18 | **CD55** | 1555950_a_at | 0.88 | 1.63 | 3.20 | 3.49 | 4.21 | 4.97 |
| 19 | **KIF14** | 236641_at | -0.12 | 0.03 | -0.62 | -1.43 | -3.01 | 4.94 |
| 20 | **ASL** | 204608_at | -0.02 | 0.70 | 1.48 | 2.35 | 2.95 | 4.92 |
| 21 | **RARB** | 208530_s_at | 2.63 | 0.25 | 3.09 | 3.21 | 3.38 | 4.92 |
| 22 | **ZNF367** | 229551_x_at | 0.52 | -0.21 | -0.09 | -1.48 | -3.08 | 4.70 |
| 23 | **BMPER** | 241986_at | -1.21 | -1.78 | -2.94 | -3.15 | -3.30 | 4.69 |
| 24 | **CFI** | 203854_at | 1.40 | 2.52 | 5.26 | 5.74 | 6.33 | 4.64 |
| 25 | **PRSS12** | 205515_at | 0.21 | 0.89 | 1.54 | 2.15 | 2.69 | 4.61 |
| 26 | **TES** | 202720_at | 0.37 | 0.76 | 1.61 | 1.96 | 2.24 | 4.54 |
| 27 | **ITGA1** | 214660_at | 1.84 | 1.32 | 3.60 | 3.95 | 4.45 | 4.54 |
| 28 | **COL6A3** | 201438_at | -0.15 | -1.11 | -2.17 | -2.51 | -3.37 | 4.47 |
| 29 | **CFI** | 1555564_a_at | 1.04 | 2.76 | 5.21 | 5.71 | 6.34 | 4.43 |
| 30 | **COLEC12** | 221019_s_at | 1.27 | 1.72 | 3.90 | 3.98 | 3.98 | 4.40 |
| 31 | **PLAT** | 201860_s_at | 0.97 | 0.69 | 2.05 | 2.35 | 2.52 | 4.34 |
| 32 | **AK091544** | 242890_at | 0.15 | 0.00 | -0.33 | -1.16 | -2.22 | 4.31 |
| 33 | **PBX1** | 212151_at | 1.12 | 0.52 | 1.99 | 2.28 | 2.45 | 4.30 |
| 34 | **ENPP2** | 209392_at | -0.15 | -0.74 | -1.65 | -2.11 | -2.31 | 4.29 |
| 35 | **CD55** | 201925_s_at | 0.78 | 2.14 | 3.68 | 3.69 | 4.37 | 4.27 |
| 36 | **SERPINA1** | 202833_s_at | 0.22 | 0.73 | 2.28 | 3.26 | 3.56 | 4.27 |
| 37 | **FRMD3** | 230645_at | -0.61 | -0.92 | -2.09 | -3.15 | -4.21 | 4.26 |
| 38 | **CMAH** | 205518_s_at | 1.30 | 0.29 | 1.70 | 2.10 | 2.94 | 4.26 |
| 39 | **HECW2** | 232080_at | -1.25 | -1.44 | -2.71 | -2.72 | -2.62 | 4.25 |
| 40 | **THBD** | 203887_s_at | 3.74 | -1.99 | 1.69 | 0.95 | -0.09 | 4.23 |
| 41 | **SERPINA1** | 211429_s_at | 0.17 | 0.55 | 1.83 | 2.79 | 2.96 | 4.21 |
| 42 | **DEPDC1** | 235545_at | -0.18 | -0.04 | -0.61 | -1.59 | -3.05 | 4.18 |
| **Supplementary Table 3 continue.** Results of the retinoic acid induced neuroblastoma cell lines from microarray data analysis | | | | | | | | |
|  |  |  | **Fold change** | |  |  |  |  |
| **N** | **Genes** | **Probe ID** | **6 hours** | **1day** | **2 days** | **3 days** | **5 days** |  |
| 43 | **DHRS3** | 202481_at | 3.70 | 0.36 | 4.17 | 4.37 | 4.76 | 4.15 |
| 44 | **ARHGAP20** | 228368_at | 1.65 | 0.71 | 3.27 | 3.82 | 4.21 | 4.15 |
| 45 | **SERPINB1** | 212268_at | 0.41 | 0.88 | 1.77 | 2.37 | 2.55 | 4.12 |
| 46 | **ANGPTL4** | 223333_s_at | 3.30 | -0.31 | 3.32 | 3.46 | 3.30 | 4.10 |
| 47 | **RARRES1** | 206392_s_at | 1.68 | 0.68 | 2.63 | 2.95 | 3.09 | 4.05 |
| 48 | **ENPP2** | 210839_s_at | -0.08 | -0.64 | -1.57 | -1.79 | -2.02 | 4.05 |
| 49 | **NCAM2** | 232390_at | 0.11 | 0.44 | 1.44 | 2.32 | 3.33 | 4.02 |
| 50 | **PRICKLE1** | 226069_at | 2.49 | 1.30 | 3.71 | 3.63 | 3.60 | 4.00 |
| 51 | **PBX1** | 212148_at | 1.28 | 0.49 | 2.23 | 2.63 | 2.81 | 3.98 |
| 52 | **GPRC5B** | 203632_s_at | 1.26 | 0.99 | 3.02 | 3.19 | 3.40 | 3.98 |
| 53 | **CYP26A1** | 206424_at | 3.04 | 2.99 | 5.95 | 5.93 | 6.33 | 3.95 |
| 54 | **PELO** | 226731_at | 1.43 | 1.28 | 3.10 | 3.50 | 4.05 | 3.93 |
| 55 | **PELO** | 1560359_at | 2.06 | 1.14 | 3.85 | 3.97 | 4.90 | 3.92 |
| 56 | N/A | 227061_at | -0.58 | -2.06 | -3.27 | -3.33 | -2.89 | 3.92 |
| 57 | **PRSS12** | 213802_at | 0.14 | 0.92 | 1.53 | 2.04 | 2.56 | 3.91 |
| 58 | **CDKN2A** | 207039_at | 0.22 | 1.22 | 2.38 | 2.96 | 3.56 | 3.88 |
| 59 | **NCAM2** | 205669_at | -0.16 | 1.20 | 2.29 | 2.76 | 3.72 | 3.87 |
| 60 | **MDK** | 209035_at | 0.17 | 0.60 | 1.07 | 1.50 | 1.89 | 3.85 |
| 61 | **GDF15** | 221577_x_at | 1.99 | 0.46 | 2.65 | 2.85 | 3.11 | 3.85 |
| 62 | **CA11** | 209726_at | 0.13 | 0.66 | 1.15 | 1.85 | 2.37 | 3.84 |
| 63 | **MPPED2** | 205413_at | 1.95 | 2.45 | 4.90 | 4.63 | 4.84 | 3.83 |
| 64 | **MCM10** | 220651_s_at | 0.23 | -0.18 | -0.47 | -1.56 | -3.08 | 3.81 |
| 65 | **IFIT2** | 226757_at | 0.88 | 0.90 | 2.83 | 3.44 | 4.29 | 3.79 |
| 66 | **FST** | 204948_s_at | -1.04 | -0.72 | -2.28 | -2.49 | -2.81 | 3.79 |
| 67 | **DTL** | 222680_s_at | 0.25 | -0.19 | -0.58 | -1.62 | -2.94 | 3.77 |
| 68 | **E2F7** | 228033_at | -0.82 | -0.33 | -1.51 | -2.38 | -3.33 | 3.75 |
| 69 | **FBN1** | 202766_s_at | -0.16 | -0.85 | -1.38 | -1.60 | -1.35 | 3.75 |
| 70 | **PION** | 222150_s_at | 0.38 | 0.93 | 2.17 | 2.45 | 3.15 | 3.74 |
| 71 | **IL8** | 211506_s_at | 1.84 | 1.08 | 3.52 | 2.91 | 2.15 | 3.73 |
| 72 | **EPHA4** | 227449_at | 0.29 | 0.33 | 1.62 | 2.26 | 3.46 | 3.72 |
| 73 | **C11orf82** | 228281_at | 0.12 | -0.26 | -0.46 | -1.47 | -2.13 | 3.71 |
| 74 | **ITGA4** | 213416_at | 0.77 | 0.97 | 2.14 | 2.20 | 2.25 | 3.71 |

| **Supplementary Table 4.** Distribution of the probes/genes according to the original source included in the model | | | | | | | |  |
| --- | --- | --- | --- | --- | --- | --- | --- | --- |
| **Gene Symbol** | **Probe ID** | **Medline Search** | **144 gene signature (Oberthuer et al., 2006)** | **55 gene signature (Asgharzadeh et al., 2006)** | **59 gene signature (Vermeulen et al., 2009)** | **32 gene signature (Fardin et al, 2010)** | **14 gene signature (Asgharzadeh et al., 2012)** | **101 genes from NB cell lines treated with retinoic acid (GSE9169)** |
|  |  |  |  |  |  |  |  |  |
| *ADCY1* | 213245_at |  |  |  |  |  |  |  |
|  |  |  |  |  |  |  |  |  |
| **AKR1C1(a)* | 216594_x_at |  |  |  |  |  |  |  |
|  |  |  |  |  |  |  |  |  |
| **AKR1C1(b)* | 204151_x_at |  |  |  |  |  |  |  |
|  |  |  |  |  |  |  |  |  |
| *ARHGEF10L* | 221656_s_at |  |  |  |  |  |  |  |
|  |  |  |  |  |  |  |  |  |
| *BTBD3* | 202946_s_at |  |  |  |  |  |  |  |
|  |  |  |  |  |  |  |  |  |
| *C9orf130* | 239799_at |  |  |  |  |  |  |  |
|  |  |  |  |  |  |  |  |  |
| *FOXP1* | 229844_at |  |  |  |  |  |  |  |
|  |  |  |  |  |  |  |  |  |
| *GFRA3* | 214479_at |  |  |  |  |  |  |  |
|  |  |  |  |  |  |  |  |  |
| **GNAI1(a)* | 227692_at |  |  |  |  |  |  |  |
|  |  |  |  |  |  |  |  |  |
| **GNAI1(b)* | 209576_at |  |  |  |  |  |  |  |
|  |  |  |  |  |  |  |  |  |
| *HOXC6* | 206858_s_at |  |  |  |  |  |  |  |
|  |  |  |  |  |  |  |  |  |
| *ING3* | 230719_at |  |  |  |  |  |  |  |
|  |  |  |  |  |  |  |  |  |
| *LOC153682* | 232794_at |  |  |  |  |  |  |  |
|  |  |  |  |  |  |  |  |  |
| *PGM2L1* | 229256_at |  |  |  |  |  |  |  |
|  |  |  |  |  |  |  |  |  |
| *PRKACB* | 202741_at |  |  |  |  |  |  |  |
|  |  |  |  |  |  |  |  |  |
| *PTPRH* | 208300_at |  |  |  |  |  |  |  |
|  |  |  |  |  |  |  |  |  |
| *RUNDC3B* | 215321_at |  |  |  |  |  |  |  |
|  |  |  |  |  |  |  |  |  |
| *SCN3A* | 210432_s_at |  |  |  |  |  |  |  |
|  |  |  |  |  |  |  |  |  |
| *SNAP91* | 204953_at |  |  |  |  |  |  |  |
|  |  |  |  |  |  |  |  |  |
| *SOX4* | 201417_at |  |  |  |  |  |  |  |
| The grey box indicates genes derive from unique source while the black box those from more sources  NB: neuroblastoma. *Two probes for the same gene. | | | | | | | | |

| **Supplementary Table 5.** Prediction ability of patient survival of neuroblastoma gene signatures | | |
| --- | --- | --- |
|  | **Seeger Dataset** | **Versteeg Dataset** |
| **SIGNATURE** | **°AUC** | **°AUC** |
| 18-gene outcome predictor | 94.63 (1) | 88.33 (1) |
| 55-gene Asgharzadeh | 94.18 (2) | 58.33 (6) |
| 14-gene Asgharzadeh | 90.36 (3) | 79.16 (3) |
| 32-gene Fardin | 84.51 (4) | 73.33 (4) |
| *144-gene Oberthuer | 83.4 (5) | 73.33 (5) |
| 59-gene Vermeulen | 82.73 (6) | 83.33 (2) |
| *127 Genes are available in the two datasets | |  |
| In parenthesis the rank position of each signature | |  |

°Area Under Curve (AUC ) was calculated by Support Vector Machines (SVM)-based area under receiver operating characteristic (ROC) curve method, utilizing ten-fold cross-validation and linear polynomial kernel for SVM

| **Supplementary Table 6.** Gene ontology of genes in the network |  |  |  |
| --- | --- | --- | --- |
| **Feature** | **FDR** | **Genes in network** | **Genes in genome** |
| activation of protein kinase A activity | 1.59E-06 | 5 | 16 |
| response to glucagon stimulus | 1.21E-05 | 5 | 33 |
| water transport | 1.21E-05 | 5 | 32 |
| G-protein signaling, coupled to cAMP nucleotide second messenger | 1.21E-05 | 6 | 70 |
| inhibition of adenylate cyclase activity by G-protein signaling pathway | 1.21E-05 | 5 | 29 |
| fluid transport | 1.21E-05 | 5 | 33 |
| cellular response to glucagon stimulus | 1.21E-05 | 5 | 33 |
| regulation of adenylate cyclase activity | 1.24E-05 | 6 | 74 |
| regulation of cyclase activity | 1.24E-05 | 6 | 75 |
| regulation of lyase activity | 1.33E-05 | 6 | 78 |
| energy reserve metabolic process | 1.33E-05 | 7 | 140 |
| cAMP-mediated signaling | 1.86E-05 | 6 | 84 |
| negative regulation of adenylate cyclase activity | 1.87E-05 | 5 | 41 |
| negative regulation of lyase activity | 1.87E-05 | 5 | 42 |
| negative regulation of cyclase activity | 1.87E-05 | 5 | 41 |
| activation of phospholipase C activity | 1.87E-05 | 6 | 88 |
| negative regulation of cAMP biosynthetic process | 1.88E-05 | 5 | 43 |
| negative regulation of cAMP metabolic process | 1.88E-05 | 5 | 43 |
| negative regulation of cyclic nucleotide biosynthetic process | 1.90E-05 | 5 | 44 |
| negative regulation of cyclic nucleotide metabolic process | 1.90E-05 | 5 | 44 |
| negative regulation of nucleotide biosynthetic process | 1.91E-05 | 5 | 45 |
| positive regulation of phospholipase C activity | 1.91E-05 | 6 | 94 |
| regulation of cAMP biosynthetic process | 1.91E-05 | 6 | 94 |
| negative regulation of nucleotide metabolic process | 1.92E-05 | 5 | 46 |
| regulation of cAMP metabolic process | 1.92E-05 | 6 | 95 |
| cAMP biosynthetic process | 1.92E-05 | 6 | 96 |
| regulation of cyclic nucleotide biosynthetic process | 1.97E-05 | 6 | 97 |
| regulation of nucleotide biosynthetic process | 2.02E-05 | 6 | 98 |
| regulation of cyclic nucleotide metabolic process | 2.21E-05 | 6 | 100 |
| positive regulation of phospholipase activity | 2.26E-05 | 6 | 101 |
| cAMP metabolic process | 2.32E-05 | 6 | 102 |
| regulation of phospholipase activity | 2.84E-05 | 6 | 106 |
| positive regulation of lipase activity | 2.84E-05 | 6 | 107 |
| cellular response to hormone stimulus | 2.84E-05 | 8 | 283 |
| regulation of nucleotide metabolic process | 3.16E-05 | 8 | 288 |
| G-protein signaling, coupled to cyclic nucleotide second messenger | 3.32E-05 | 6 | 111 |
| cyclic nucleotide biosynthetic process | 3.60E-05 | 6 | 113 |
| cyclic nucleotide metabolic process | 6.23E-05 | 6 | 125 |
| regulation of lipase activity | 6.23E-05 | 6 | 125 |
| fibroblast growth factor receptor signaling pathway | 7.15E-05 | 6 | 129 |
| cyclic-nucleotide-mediated signaling | 7.15E-05 | 6 | 129 |
| nucleoside monophosphate biosynthetic process | 7.65E-05 | 6 | 131 |
| cellular response to fibroblast growth factor stimulus | 9.12E-05 | 6 | 136 |
| response to fibroblast growth factor stimulus | 9.12E-05 | 6 | 136 |
| epidermal growth factor receptor signaling pathway | 1.44E-04 | 6 | 148 |
| nucleoside monophosphate metabolic process | 1.44E-04 | 6 | 148 |
| purine nucleotide biosynthetic process | 1.65E-04 | 6 | 152 |
| purine-containing compound biosynthetic process | 2.80E-04 | 6 | 167 |
| energy derivation by oxidation of organic compounds | 3.10E-04 | 7 | 276 |
| nucleotide biosynthetic process | 3.91E-04 | 6 | 178 |
| activation of adenylate cyclase activity | 4.99E-04 | 4 | 43 |
| aldo-keto reductase (NADP) activity | 5.08E-04 | 3 | 12 |
| adenylate cyclase activity | 5.08E-04 | 3 | 12 |
| positive regulation of adenylate cyclase activity | 5.17E-04 | 4 | 44 |
| positive regulation of cyclase activity | 5.57E-04 | 4 | 45 |
| positive regulation of lyase activity | 6.53E-04 | 4 | 47 |
| nucleobase-containing compound biosynthetic process | 6.96E-04 | 6 | 201 |
| phosphorus-oxygen lyase activity | 7.41E-04 | 3 | 14 |
| oxidoreductase activity, acting on the CH-CH group of donors, NAD or NADP as acceptor | 7.41E-04 | 3 | 14 |
| cellular response to growth factor stimulus | 7.41E-04 | 6 | 204 |
| second-messenger-mediated signaling | 8.61E-04 | 6 | 211 |
| cyclase activity | 8.67E-04 | 3 | 15 |
| oxidoreductase activity, acting on paired donors, with incorporation or reduction of molecular oxygen, NADH or NADPH as one donor, and incorporation of one atom of oxygen | 8.67E-04 | 3 | 15 |
| response to growth factor stimulus | 8.67E-04 | 6 | 212 |
| nerve growth factor receptor signaling pathway | 9.26E-04 | 6 | 216 |
| heterocycle biosynthetic process | 1.10E-03 | 6 | 223 |
| regulation of hormone levels | 1.42E-03 | 6 | 234 |
| positive regulation of cAMP biosynthetic process | 1.52E-03 | 4 | 61 |
| positive regulation of cAMP metabolic process | 1.52E-03 | 4 | 61 |
| positive regulation of cyclic nucleotide biosynthetic process | 1.60E-03 | 4 | 62 |
| GTPase activity | 1.62E-03 | 5 | 137 |
| positive regulation of nucleotide biosynthetic process | 1.66E-03 | 4 | 63 |
| positive regulation of cyclic nucleotide metabolic process | 1.74E-03 | 4 | 64 |
| positive regulation of nucleotide metabolic process | 1.95E-03 | 4 | 66 |
| regulation of adenylate cyclase activity involved in G-protein signaling pathway | 2.78E-03 | 3 | 23 |
| positive regulation of adenylate cyclase activity by G-protein signaling pathway | 2.78E-03 | 3 | 23 |
| activation of adenylate cyclase activity by G-protein signaling pathway | 2.78E-03 | 3 | 23 |
| sequence-specific DNA binding RNA polymerase II transcription factor activity | 4.21E-03 | 5 | 170 |
| sequence-specific distal enhancer binding RNA polymerase II transcription factor activity | 4.76E-03 | 4 | 84 |
| C21-steroid hormone metabolic process | 4.91E-03 | 3 | 28 |
| drug metabolic process | 5.92E-03 | 3 | 30 |
| activation of protein kinase activity | 5.92E-03 | 5 | 184 |
| oxidoreductase activity, acting on the CH-CH group of donors | 8.58E-03 | 3 | 34 |
| cellular response to peptide hormone stimulus | 1.34E-02 | 5 | 220 |
| response to peptide hormone stimulus | 1.64E-02 | 5 | 230 |
| glucose metabolic process | 2.72E-02 | 4 | 134 |
| oxidoreductase activity, acting on NADH or NADPH, quinone or similar compound as acceptor | 3.49E-02 | 3 | 55 |
| monooxygenase activity | 3.84E-02 | 3 | 57 |
| DNA binding, bending | 4.00E-02 | 3 | 58 |
| smooth muscle tissue development | 4.15E-02 | 2 | 11 |
| histone H2A acetylation | 4.92E-02 | 2 | 12 |
| cellular hormone metabolic process | 5.13E-02 | 3 | 64 |
| isomerase activity | 5.13E-02 | 3 | 64 |
| hexose metabolic process | 5.16E-02 | 4 | 162 |
| oxidoreductase activity, acting on the CH-OH group of donors, NAD or NADP as acceptor | 6.27E-02 | 3 | 69 |
| H4/H2A histone acetyltransferase complex | 6.29E-02 | 2 | 14 |
| NuA4 histone acetyltransferase complex | 6.29E-02 | 2 | 14 |
| steroid dehydrogenase activity, acting on the CH-OH group of donors, NAD or NADP as acceptor | 6.29E-02 | 2 | 14 |
| oxidoreductase activity, acting on paired donors, with incorporation or reduction of molecular oxygen | 6.54E-02 | 3 | 71 |
| positive regulation of mesenchymal cell proliferation | 7.03E-02 | 2 | 15 |
| lung epithelium development | 7.03E-02 | 2 | 15 |
| extrinsic to membrane | 7.18E-02 | 3 | 74 |
| oxidoreductase activity, acting on CH-OH group of donors | 7.39E-02 | 3 | 75 |
| lyase activity | 7.61E-02 | 3 | 76 |
| G-protein beta/gamma-subunit complex binding | 7.72E-02 | 2 | 16 |
| glycogen catabolic process | 8.57E-02 | 2 | 17 |
| steroid dehydrogenase activity | 8.57E-02 | 2 | 17 |
| oxidoreductase activity, acting on NADH or NADPH | 8.96E-02 | 3 | 82 |
| GTPase regulator activity | 8.96E-02 | 4 | 196 |
| prostaglandin metabolic process | 8.96E-02 | 2 | 18 |
| cellular polysaccharide catabolic process | 8.96E-02 | 2 | 18 |
| glucan catabolic process | 8.96E-02 | 2 | 18 |
| monosaccharide metabolic process | 8.96E-02 | 4 | 196 |
| lung morphogenesis | 8.96E-02 | 2 | 18 |
| regulation of mesenchymal cell proliferation | 8.96E-02 | 2 | 18 |
| nucleoside-triphosphatase regulator activity | 9.64E-02 | 4 | 202 |

FDR: False Discovery Rate
